# Azobenzene dye-coupled quadruply hydrogen-bonding modules as colorimetric indicators for supramolecular interactions

**DOI:** 10.3762/bjoc.8.55

**Published:** 2012-04-02

**Authors:** Yagang Zhang, Steven C Zimmerman

**Affiliations:** 1 Department of Chemistry, 600 South Mathews Avenue, University of Illinois, Urbana, IL 61801, USA

**Keywords:** azobenzene dye, colorimetric indicators, 7-deazaguanine urea (DeUG), 2,7-diamido-1,8-naphthyridine (DAN), polymer, quadruply hydrogen bonding, supramolecular

## Abstract

The facile coupling of azobenzene dyes to the quadruply hydrogen-bonding modules 2,7-diamido-1,8-naphthyridine (DAN) and 7-deazaguanine urea (DeUG) is described. The coupling of azobenzene dye **2** to mono-amido DAN units **4**, **7**, and **9** was effected by classic 4-(dimethylamino)pyridine (DMAP)-catalyzed peptide synthesis with *N*-(3-dimethylaminopropyl)-*N*’-ethyl carbodiimide hydrochloride (EDC) as activating agent, affording the respective amide products **5**, **8**, and **10** in 60–71% yield. The amide linkage was formed through either the aliphatic or aromatic ester group of **2**, allowing both the flexibility and absorption maximum to be tuned. Azobenzene dye **1** was coupled to the DeUG unit **11** by Steglich esterification to afford the product amide **12** in 35% yield. Alternatively, azobenzene dye **16** underwent a room-temperature copper-catalyzed azide–alkyne Huisgen cycloaddition with DeUG alkyne **17** to give triazole **18** in 71% yield. Azobenzene coupled DAN modules **5**, **8**, and **10** are bright orange–red in color, and azobenzene coupled DeUG modules **12** and **18** are orange–yellow in color. Azobenzene coupled DAN and DeUG modules were successfully used as colorimetric indicators for specific DAN–DeUG and DAN–UPy (2-ureido-4(1*H)*-pyrimidone) quadruply hydrogen-bonding interactions.

## Introduction

Lehn’s pioneering studies [1] have advanced supramolecular chemistry to the point where complex hierarchical self-assembled [2] and even dynamically assembled structures are routinely described [3]. Noncovalent hydrogen bonding, electrostatic interactions, π–π stacking and metal coordination have been used alone and in concert to assemble a broad range of building blocks, from small molecules [4–7] to polymers [8–9] including dendrimers [10–11]. Among these noncovalent interactions, hydrogen bonding is especially useful, not only because of its predictability both in terms of strength and geometry, but also because of its intrinsically dynamic and reversible nature. Of the hydrogen-bonding units developed for assembly, those that feature multiple hydrogen bonding sites are particularly desirable because they usually pair with high affinity and high fidelity [12]. High-affinity hydrogen-bonding units have found particular applications in supramolecular polymers [13–14], in which the presence of fewer hydrogen bonds means that the desired assemblies are not achieved, or results in polymers of lower molecular weight. The utility of these units is further demonstrated in the broad range of supramolecular architectures that have emerged and the materials to which these units have been attached, including dendrimers [15–17], polymer chain-ends [18–20] and polymer side-chains [21–22], modified polymeric materials, functionalized nano-structures [23] and surfaces [24].

**Figure 1:**
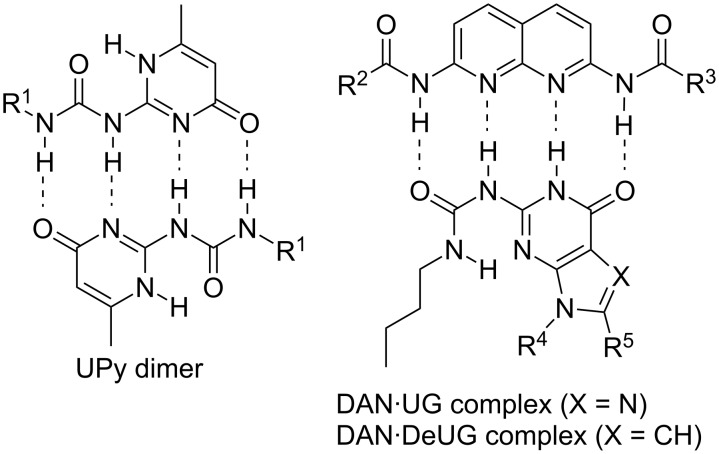
Chemical structures of UPy dimer and DAN complexes with UG and DeUG.

Of the numerous supramolecular coupling agents developed those that pair using quadruply hydrogen bonding have arguably received the most attention in the context of supramolecular polymer chemistry. In particular, the highly stable UPy dimers developed by Meijer and Sijbesma [9,14,25] and the high-stability and high-fidelity DAN·UG heterodimers developed in our laboratory [26–27] are appealing because beyond the stable complexes that they form, they are synthetically quite accessible (Figure 1) [28]. Indeed, several syntheses of the DAN unit are now available [29–34]. The original UG unit contains a labile nucleoside unit, but a DeUG unit [35] and more recently a DeUG module bearing a range of synthetic handles for further elaboration [36] were both reported with more scalable syntheses. With regard to applications, the DAN·UG (DeUG) heterodimer has been used to drive the formation of: (1) polymer blends [37], (2) a supramolecular multiblock copolymer with a high propensity for alteration [38], and (3) a supramolecular ABC triblock polymer [39]. Further, a structure–property relationship has been developed for DAN·UG-based supramolecular-network polymer blends [40], and a redox-active eDAN unit was described wherein a >10^4^-fold drop in binding affinity occurred upon reversible oxidation [41].

**Figure 2:**
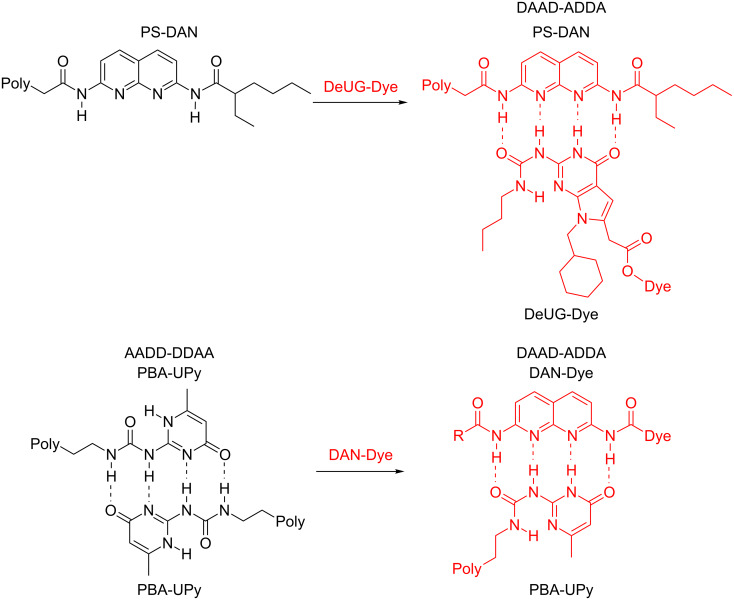
Illustration of the use of DeUG-Dye and DAN-Dye as colorimetric indicators for supramolecular interactions through specific quadruply hydrogen-bonding interactions. Top: DeUG-Dye interacts with DAN modified polystyrene (PS). Bottom: DAN-Dye interacts with UPy modified poly-butyl acrylate (PBA).

Herein, we extend the chemistry of the heterocyclic hydrogen bonding units (DAN and DeUG) by their coupling to azobenzene dyes allowing them to serve as colorimetric indicators for supramolecular interactions. Beyond reporting the straightforward coupling of DAN and DeUG to azobenzene dyes, we show that the dye-recognition-unit conjugates act as selective polymer colorants (Figure 2). The development of a recognition unit opens the possibility of their use as a colorimetric handle for monitoring and investigating quadruply hydrogen-bonding interactions at molecular levels and in materials applications.

## Results and Discussion

The azobenzene units were chosen because they are widely used as dyes and exhibit a range of vivid colors. Furthermore, the application of azobenzenes in chemistry is quite broad and includes their use as switches [42], in nonlinear optics [43], sensing devices [44], and in nanostructured films for optical storage [45]. Although this work does not focus on switching, the ability to synthesize these recognition units containing azobenzene units opens up the possibility to turn hydrogen bonding on and off. With regard to synthesis, aromatic azo compounds are commonly prepared by an electrophilic substitution reaction, the best partners being an electron-rich aromatic ring and an aryl diazonium cation.

**Scheme 1:**
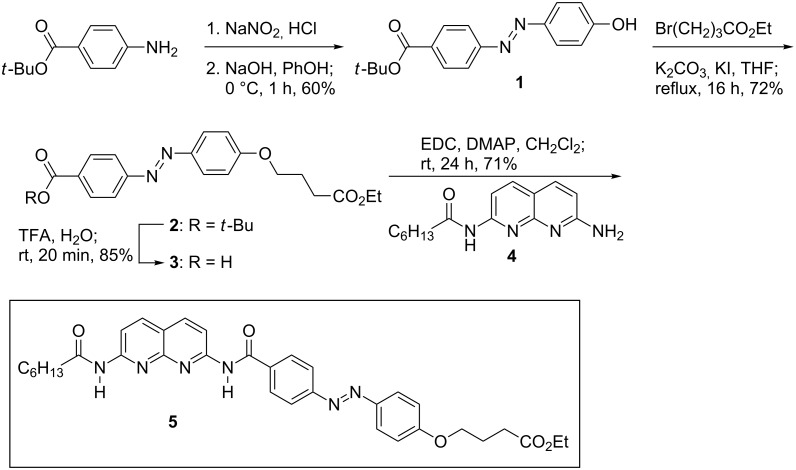
Synthesis of azobenzene-dye-coupled DAN **5**.

The synthesis of azobenzene-dye-coupled DAN **5** began with 4-aminobenzoic acid *tert*-butyl ester (Scheme 1). Diazotization and coupling with phenol afforded **1** in 60% yield, which was comparable to the reported synthesis [46]. Compound **1** was alkylated with ethyl 4-bromobutyrate to give **2** in 72% yield [47]. Acid-catalyzed deprotection of **2** afforded carboxylic acid **3** in 85% yield [48]. The coupling of **3** with the mono-heptanamide of 2,7-diamino-1,8-naphthyridine (DAN **4**) was effected by using a standard peptide-coupling method. Thus, 1-(3-dimethylaminopropyl)-3-ethylcarbodiimide hydrochloride (EDC) was used as an activating agent and 4-(dimethylamino)pyridine (DMAP) was used as a catalyst [49]. Azobenzene–DAN conjugate **5** was obtained in 71% yield as an orange–red solid following chromatography.

**Scheme 2:**
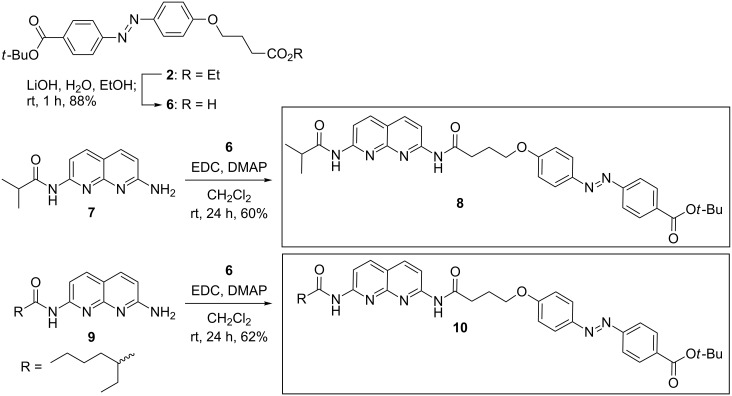
Synthesis of azobenzene-dye-coupled DAN **8** and **10**.

Deprotection of azobenzene **2** under mildly basic conditions was also investigated, which afforded compound **6** in 88% yield (Scheme 2). The coupling of monoamide derivatives of 2,7-diamido-1,8-naphthyridine (DAN), i.e., **7** and **9** with **6** under similar coupling conditions, yielded **8** and **10** in 60% and 62% yield, respectively. All three azobenzene-coupled DAN modules were vivid orange–red in color and had excellent solubility in a range of nonpolar organic solvents.

**Scheme 3:**
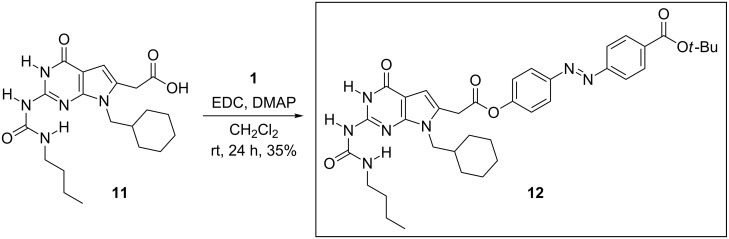
Synthesis of azobenzene dye-coupled DeUG **12**.

The initial attempt to couple DeUG unit **11** to azobenzene dye **1** utilized the established Steglich esterification procedure [50] with DMAP as the catalyst [51]. Thus, DeUG carboxylic acid **11** was coupled with **1** by using EDC and DMAP in methylene chloride, and the orange–yellow product **12** was isolated in a relatively low 35% yield (Scheme 3). No attempt was made to optimize the coupling conditions; instead, attention was turned to the possibility of coupling the partners by using the copper-catalyzed azide–alkyne Huisgen cycloaddition (click reaction).

**Scheme 4:**
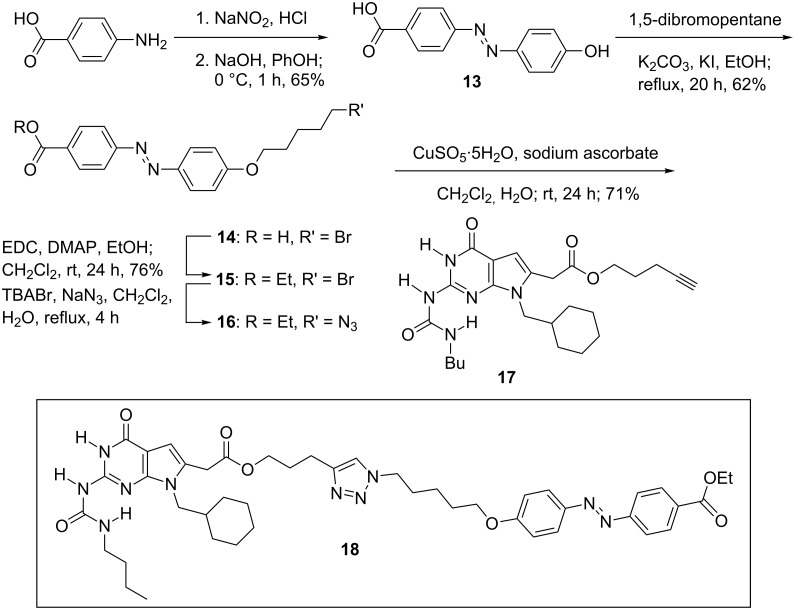
Synthesis of azobenzene dye-coupled DeUG **18**.

The click approach began with the readily available and inexpensive starting material, 4-aminobenzoic acid (Scheme 4), which was diazotized and treated with phenol to afford **13** in 65% yield, comparable to published procedures [52–54]. To install the azide functionality, **13** was treated with 1,5-dibromopentane and potassium carbonate to afford bromide **14** in 62% yield. Because of its poor solubility, **14** was esterified to afford ethyl ester **15** in 76% yield. Treatment of **15** with sodium azide in the presence of tetrabutyl ammonium bromide as a phase-transfer reagent produced crude **16**, which was used directly in the next step without purification. Coupling of azide **16** with the known **17** [34] was successfully effected under standard conditions for the copper-catalyzed azide–alkyne cycloaddition [55]. Azobenzene dye-coupled DeUG module **18** was obtained as an orange–yellow solid in 71% yield. The product was assigned as the 1,4-substituted triazole by analogy to that seen in other such cycloaddition reactions [56].

**Figure 3:**
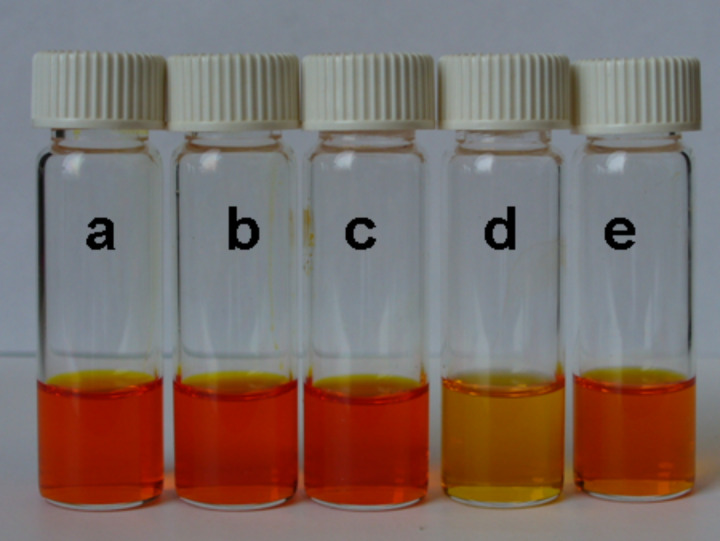
Solution (20 mmol) of azobenzene-dye-coupled DAN and DeUG in CH_2_Cl_2_; a = compound **5**, b = compound **8**, c = compound **10**, d = compound **12**, e = compound **18**.

Azobenzene-coupled DAN modules **5**, **8**, and **10** are bright orange–red in color and azobenzene-coupled DeUG modules **12** and **18** are orange–yellow in color (Figure 3). All of the azobenzene coupled DAN and DeUG modules have excellent solubility in nonpolar organic solvents, wherein their supramolecular recognition is most effective.

**Figure 4:**
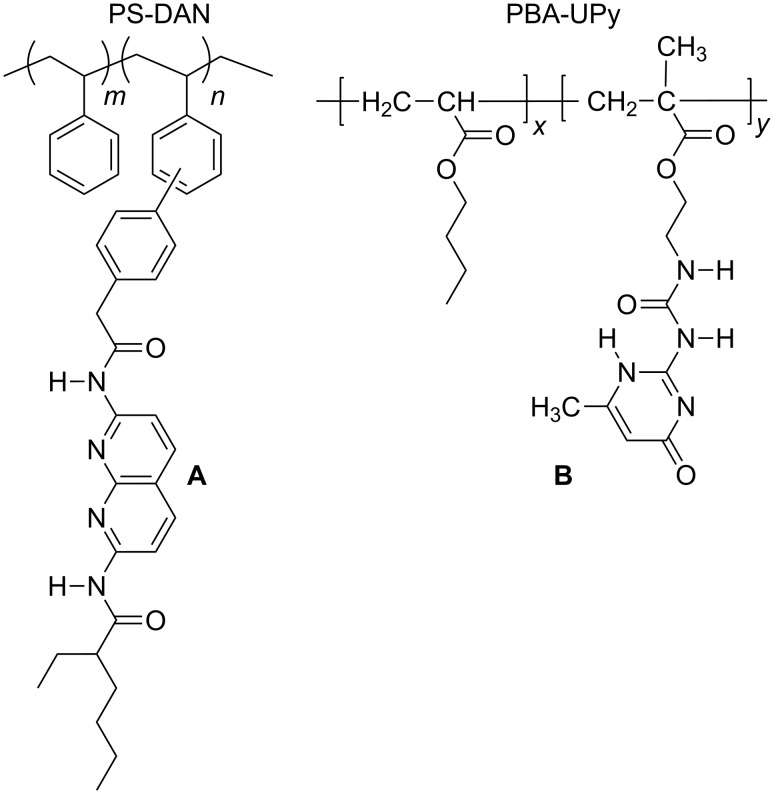
Structure of DAN-modified PS and Upy-modified PBA.

To study the ability of the DAN and DeUG-coupled azobenzene dyes to engage in quadruply hydrogen-bonding interactions and demonstrate the possibility of using these compounds as colorimetric indicators, two types of polymers bearing DAN and UPy were used (Figure 4). The DAN modified polystyrene (PS-DAN) was a gift from Dr. Cyrus Anderson and its synthesis will be published elsewhere. Commercially available polystyrene (PS) was used as a control polymer and purified by dissolving in CH_2_Cl_2_ and precipitating out with MeOH.

PS and PS-DAN were characterized by SEC with THF as eluent, with PS molecular weight standards. The loading of the DAN unit was determined by ^1^H NMR (Table 1). Poly(butyl acrylate) (PBA) and 2-ureido-4(1*H*)-pyrimidone (UPy) modified poly(butyl acrylate) (PBA-UPy) were prepared and characterized by THF SEC against PS standards according to a known procedure [57]. The loading of the UPy units, determined by ^1^H NMR, is listed in Table 1.

**Table 1:** Molecular weight, molecular weight distribution and mol % loading of DAN modified PS and UPy modified PBA.

entry	polymer	*M*_w_ (kDa)	*M*_n_ (kDa)	PDI

A0	PS	138	69	2.0
A1	PS-DAN (2.0 mol %)	148	114	1.3
A2	PS-DAN (5.0 mol %)	131	73	1.8
B0	PBA	84	38	2.2
B1	PBA-UPy (2.6 mol %)	84	38	2.2
B2	PBA-UPy (4.1 mol %)	80	38	2.1
B3	PBA-UPy (7.1 mol %)	110	55	2.0

**Figure 5:**
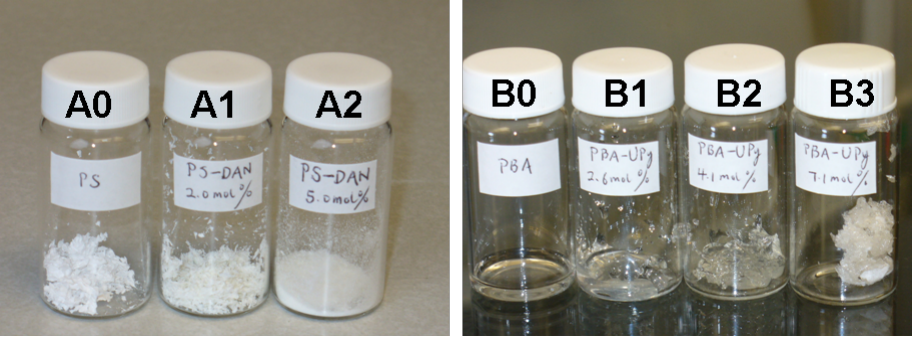
Physical appearance of DAN-modified PS and UPy-modified PBA. Left: A0 = PS, A1 = PS-DAN 2.0 mol %, A2 = PS-DAN 5.0 mol %. Right: B0 = PBA, B1 = PBA-UPy 2.6 mol %, B2 = PBA-UPy 4.1 mol %, B3 = PBA-UPy 7.1 mol %.

PS and DAN-modified PS were obtained as white powders whereas PBA and UPy-modified PBA ranged from viscous liquids to gels depending on the loading of UPy groups on the polymer backbone (Figure 5). The supramolecular coupling of DAN and DeUG units and DAN and UPy units has been well studied. Thus, the association constant (*K*_assoc_) for the DAN–DeUG heterocomplex was measured as *K*_assoc_ ≈ 10^8^ M^−1^ [36], whereas for the DAN–UPy heterocomplex *K*_assoc_ = 10^6^ M^−1^ [58–60].

**Figure 6:**
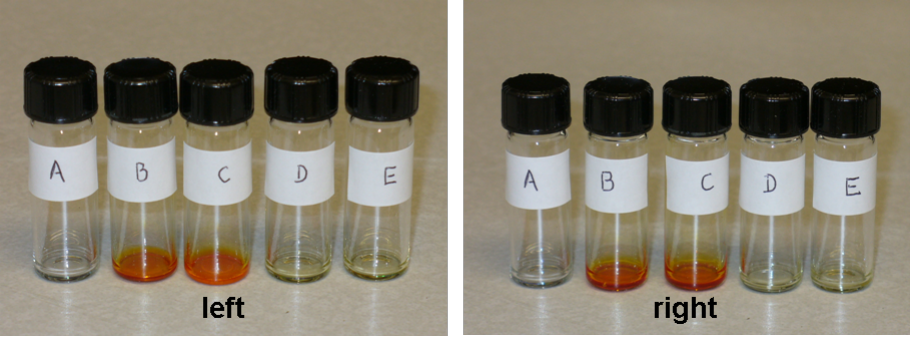
Color change after the interaction of azo-benzene dye-coupled DeUG modules with different DAN modified PS, followed by washing. Left: (A) PS with compound **12**, (B) PS-DAN (2.0 mol %) with compound **12**, (C) PS-DAN (2.0 mol %) with compound **18**, (D) PS-DAN (2.0 mol %) with compound **2**, (E) PS-DAN (2.0 mol %) with compound **15**. Right: (A) PS with compound **18**, (B) PS-DAN (5.0 mol %) with compound **12**, (C) PS-DAN (5.0 mol %) with compound **18**, (D) PS-DAN (5.0 mol %) with compound **2**, (E) PS-DAN (5.0 mol %) with compound **15**.

Whether these complex stabilities allow for the selective coloration of the functional polymers was examined by mixing CH_2_Cl_2_ solutions of polymer and dye-coupled recognition units, evaporation of the solvent, and repeated washing of the residue with CH_2_Cl_2_–hexanes in an attempt to remove the color. These conditions were selected because they were effective at removing dye from the unfunctionalized polymer. Thus, as seen in Figure 6 (left panel, vial A), PS did not retain DeUG-Dye compound **12** after washing. However, PS-DAN (2 mol %) retained both compound **12** and **18** (vial B and C, respectively), showing intense coloration. The designed supramolecular recognition (DAN–DeUG heterocomplex) was shown to be the key factor by a control experiment featuring PS-DAN and azobenzene dyes **2** and **15**, both of which lack the DeUG unit. As seen in Figure 6 (left panel, vial D and E), the dye was washed away from the polymer. Not surprisingly the 5 mol % PS-DAN performed at least as well in the same series of experiments (Figure 6, right panel). The overall approach is meant to illustrate the strength and specificity of the recognition process, not to provide a quantitative method. Thus, although the washing procedure with a mixed nonpolar solvent (CH_2_Cl_2_–hexanes) proved to be effective in removing nonspecifically associated compounds, over 50% of the polymer was also lost during the multiple washings.

**Figure 7:**
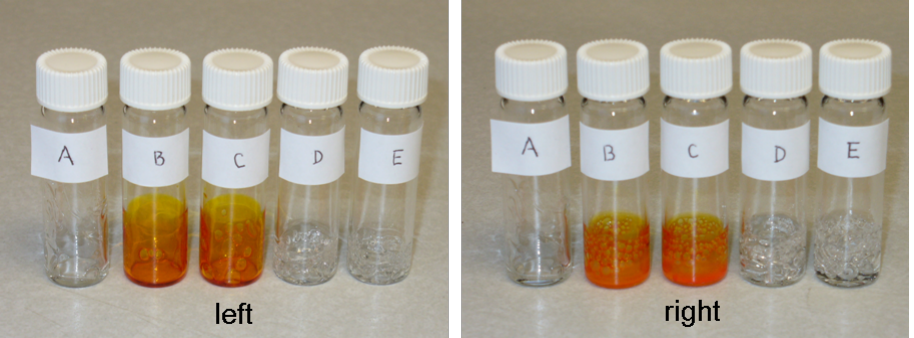
Color change after the interaction of azobenzene dye-coupled DAN modules with different UPy-modified PBA, followed by washing. Left: (A) PBA with compound **5**, (B) PBA-UPy (4.1 mol %) with compound **5**, (C) PBA-UPy (4.1 mol %) with compound **8**, (D) PBA-UPy (4.1 mol %) with compound **2** (E) PBA-UPy (4.1 mol %) with compound **15**. Right: (A) PBA with compound **5**, (B) PBA-UPy (7.1 mol %) with compound **8**, (C) PBA-UPy (7.1 mol %) with compound **10**, (D) PBA-UPy (7.1 mol %) with compound **2**, (E) PBA-UPy (7.1 mol %) with compound **15**.

The generality of this recognition process was demonstrated by observing the same trend with DAN-Dyes **5**, **8** and **10** and UPy-modified PBA (Figure 7). Thus, after mixing of DAN-Dyes **5**, **8** and **10** with UPy modified PBA (both 4.1 mol % and 7.1 mol %) the polymers became highly colored. However, after being washed repeatedly with CH_2_Cl_2_–hexanes, unfunctionalized PBA lost its color (Figure 7, left and right panels, vial A) whereas PBA-UPy retained an intense orange color (Figure 7 left and right panels, vials B and C). Control studies with PBA-UPy (both 4.1 mol % and 7.1 mol %) mixed with azobenzene dye **2** and **15** lacking a DAN-recognition unit, showed a complete loss of color following extensive washing (Figure 7 left and right panels, vials D and E).

## Conclusion

In conclusion, we have demonstrated straightforward and scalable syntheses of azobenzene dye-coupled quadruply hydrogen-bonding recognition modules. Specifically, the 2,7-diamido-1,8-naphthyridine (DAN) unit was linked to azobenzene dyes through one of its amide groups, giving compounds **5**, **8**, and **10**, and the 7-deazaguanine urea (DeUG) unit was linked to an azobenzene dye by a Steglich esterification, giving **12**, or by the copper-catalyzed azide–alkyne cycloaddition (click reaction) to give **18**. The synthesis provides access to highly colored recognition units that may serve as useful probes of recognition events. In this work we have successfully demonstrated that these units colored two polymers, PBA and PS, but only when they contain the complementary recognition units along their backbone.

## Experimental

### General

With the exception of 1-(3-dimethylaminopropyl)-3-ethylcarbodiimide hydrochloride (EDC), which was purchased from Advanced ChemTech and used as received, all other chemicals were purchased from Sigma-Aldrich and used without further purification. Compounds **4**, **7**, and **9** were synthesized according to the published procedures [34], as were compounds **11** and **17** [36]. Solvents were reagent grade and used without further purification except as follows: Tetrahydrofuran (THF) was distilled from sodium benzophenone ketyl immediately prior to use. Methylene chloride (CH_2_Cl_2_) was obtained from an MB-SPS Solvent Purification System and stored over 4 Å molecular sieves. All reactions were carried out under a dry nitrogen atmosphere, except for the preparation of compounds **1** and **13**. Ambient or room temperature refers to 25 ± 3 °C.

### Representative procedure for testing polymer coloration

One representative example each of coloration experiments with (a) DeUG-Dye **18** with PS-DAN and (b) DAN-Dye **5** with PBA-UPy: (a) 0.30 g PS-DAN (5.0 mol %) was dissolved in 25 mL of CH_2_Cl_2_, then 5.0 mL of a solution of DeUG-Dye **18** in CH_2_Cl_2_ (20 mM) was added and the mixture was stirred for 3 h. The solvent was removed in vacuo. The resulting solid was washed with 0.2% CH_2_Cl_2_–hexanes (v/v) (10 × 12 mL). Each washing was carried out in a 20 mL scintillation vial under stirring for 40 min. After settling of the polymer, the washing solvent was decanted and removed with a pipette. The final polymer was transferred into a small vial and dried at room temperature. (b) To a solution of 0.30 g PBA-UPy (4.1 mol %) in 25 mL CH_2_Cl_2_ was added 5.0 mL of a solution of DAN-Dye **5** in CH_2_Cl_2_ (20 mM) and the mixture was stirred for 3 h. The solvent was removed in vacuo. The resulting solid was washed with 1% CH_2_Cl_2_–hexanes (v/v) (10 × 12 mL). Each washing was carried out in a 20 mL scintillation vial under stirring for 40 min. After settling of the polymer, the washing solvent was decanted and removed with a pipette. The final polymer was transferred into a small vial and dried at room temperature.

## Supporting Information

File 1 General experimental procedures, detailed synthetic procedures and characterization data.
