# Supplementary material for: Azobenzene dye-coupled quadruply hydrogen-bonding modules as colorimetric indicators for supramolecular interactions
Source: Beilstein J Org Chem. 2012 Apr 2;8:486–95. doi: 10.3762/bjoc.8.55 (PMC3326628; doi:10.3762/bjoc.8.55)
Supplement: File 1 — General experimental procedures, detailed synthetic procedures and characterization data. [file Beilstein_J_Org_Chem-08-486-s001.pdf]

# Supporting Information

for

# Azobenzene dye-coupled quadruply hydrogen-bonding modules as colorimetric indicators for supramolecular interactions

Yagang Zhang and Steven C. Zimmerman*

Address: Department of Chemistry, 600 South Mathews Avenue, University of Illinois, Urbana, IL 61801, USA

Email: Steven C. Zimmerman* - sczimmer@illinois.edu

*Corresponding author

## General experimental procedures, detailed synthetic procedures and characterization data

## Table of contents

**Experimental section**

**Additional general methods** ____ S2

**Synthetic procedures** ____ S3

**$^1$H and $^{13}$C NMR spectra**

**Compound 1** ____ S10

**Compound 2** ____ S11

**Compound 3** ____ S12

**Compound 5** ____ S13

**Compound 6** ____ S14

**Compound 8** ____ S15

**Compound 10** ____ S16

**Compound 12** S17
**Compound 13** S18
**Compound 14** S19
**Compound 15** S20
**Compound 18** S21
**Elemental composition reports of HR-ESI-MS** S22
**References** S28

## Experimental section

### Additional general methods

Analytical thin-layer chromatography (TLC) was performed by using 0.2 mm silica 60 coated, plastic plates with $F_{254}$ indicator purchased from EM Science. Column chromatography was performed by using Ultra Pure SiliaFlash® P60 230–400 mesh (40–63 µm) silica gel ($SiO_2$) with fluorescence indicator green 254 nm (Fluka) following the general procedure reported by Still and co-workers [1]. Analytical size-exclusion chromatography (SEC) was performed in THF with a flow-rate of 1 mL $min^{-1}$ (30 °C) using Waters auto sampler, pump, photodiode array and refractive-index detector outfitted with Waters Styragel columns (HR5, HR4, HR3, HR1). Molecular weight was determined using conventional calibration against polystyrene standards.

$^{1}H$ NMR spectra were acquired on a Varian Unity 500 MHz ($^{13}C$, 126 MHz) spectrometer. $^{1}H$ NMR chemical shifts (δ) are reported in parts per million (ppm) and are referenced to the residual solvent peak at 7.26 ppm for $CDCl_3$, 2.50 ppm for DMSO-$d_6$ and 3.31 ppm for $CD_3OD$. $^{1}H$ NMR coupling constants (*J*) are reported in hertz (Hz). Several of the aromatic protons in the azobenzene units are listed as doublets although additional coupling and broadening was seen in most of the aromatic resonances. $^{13}C$ NMR chemical shifts are reported in ppm and were referenced to the residual solvent peak at 77.16 ppm for $CDCl_3$, 39.52 ppm for DMSO-$d_6$ and 49.00 ppm for $CD_3OD$. All NMR spectra are original spectra obtained from the NMR instrument and then scanned. Mass spectra were obtained on Micromass Q-Tof Ultima (HR-ESI) and Micromass Quattro (LR-ESI) instruments. Melting points were determined by using a Thomas-Hoover melting point apparatus and are uncorrected.

## Synthetic procedures

### *tert*-Butyl 4-[(4-hydroxyphenyl)diazenyl]benzoate (1)

To a stirred suspension of 4-aminobenzoic acid *tert*-butyl ester (2.40 g, 12.0 mmol) in 30 mL of a 2 M aqueous hydrochloric acid solution cooled in a 0 °C ice bath, was added dropwise 5 mL of an aqueous solution of $NaNO_2$ (900 mg, 13.0 mmol). The solution was stirred for 8 min and diluted with ice-cold MeOH (50 mL). The resultant yellow solution was added slowly to a solution of phenol (1.17 g, 12.4 mmol) and NaOH (960 mg, 24.0 mmol) in 14 mL MeOH cooled to 0 °C in an ice bath. The solution was stirred for 1 h and a concentrated aqueous solution of HCl was added until pH 1 to 2 was attained. The precipitate was filtered off and washed with an excess of water. The crude product was crystallized from EtOH–$H_2O$ (1:10, *v*/*v*) to afford 2.23 g (60%) of **1** as an orange solid: mp 144-145 °C; $^1$H NMR (500 MHz, $CDCl_3$) δ 8.12 (d, *J* = 8.5, 2H), 7.92 (d, *J* = 9.0, 2H), 7.88 (d, *J* = 8.5, 2H), 6.97 (d, *J* = 9.0, 2H), 5.41 (s, 1H), 1.62 (s, 9H); $^{13}$C NMR (126 MHz, $CDCl_3$) δ 165.5, 158.9, 155.2, 147.4, 133.4, 130.6, 125.5, 122.4, 116.0, 81.6, 28.4, 28.3; ESI-HR-MS calcd for $(C_{17}H_{18}N_2O_3+H)^+$ 299.1396, found 299.1395.

### *tert*-Butyl 4-{[4-(4-ethoxy-4-oxobutoxy)phenyl]diazenyl}benzoate (2)

A mixture of **1** (1.00 g, 3.35 mmol), ethyl 4-bromobutyrate (2.75 g, 13.4 mmol), potassium carbonate (1.39 g, 10.1 mmol), and potassium iodide (500 mg, 0.301 mmol) in 50 mL of THF was stirred and heated under reflux for 16 h. The mixture was cooled, concentrated in vacuo, and dissolved in ca. 35 mL of $CH_2Cl_2$. The solution was washed with 30 mL of a 2.0 M aqueous solution of HCl and 30 mL of brine. The organic layer was concentrated in vacuo and the residue purified by silica gel column chromatography with MeOH–$CH_2Cl_2$ (1:99 *v*/*v*, $R_f$ = 0.72) to afford 995 mg (72%) of **2** as an orange solid: mp 108-109 °C; $^1$H NMR (500 MHz, $CDCl_3$) δ 8.11 (d, *J* = 8.5, 2H), 7.93 (d, *J* = 9.0, 2H), 7.88 (d, *J* = 8.5, 2H), 7.00 (d, *J* = 9.0, 2H), 4.16 (q, *J* = 7.0, 2H), 4.10 (t, *J* = 6.0, 2H), 2.54 (t, *J* = 7.0, 2H), 2.14-2.18 (m, 2H), 1.62 (s, 9H), 1.26 (t, *J* = 7.0, 3H); $^{13}$C NMR (126 MHz, $CDCl_3$) δ 173.2, 165.4, 162.0, 155.2, 147.2, 133.3, 130.5, 125.3, 122.3, 115.0, 114.8, 81.5, 67.3, 60.7, 30.8, 28.5, 28.4, 28.3, 24.7, 14.4, 14.3; ESI-HR-MS calcd for $(C_{23}H_{28}N_2O_5+H)^+$ 413.2076, found 413.2079.

### 4-{[4-(4-Ethoxy-4-oxobutoxy)phenyl]diazenyl}benzoic acid (3)

Trifluoroacetic acid (4 mL, 52 mmol) was added to **2** (600 mg, 1.45 mmol). The mixture was stirred for 20 min. The excess trifluoroacetic acid was removed in vacuo and 2 mL acetone was

added. The solid was filtered off and was purified by silica gel column chromatography with MeOH-$CH_2Cl_2$ (5:95 *v*/*v*, $R_f$ = 0.25) to afford 440 mg (85%) of **3** as an orange solid: mp 211-212 °C; $^1$H NMR (500 MHz, DMSO-$d_6$) δ 13.20 (bs, 1H), 8.10 (d, *J* = 9.0, 2H), 7.91 (d, *J* = 9.5, 2H), 7.89 (d, *J* = 8.5, 2H), 7.13 (d, *J* = 9.0, 2H), 4.11 (t, *J* = 6.5, 2H), 4.07 (q, *J* = 7.0, 2H), 2.51 (t, *J* = 2.0, 2H), 1.99-2.02 (m, 2H), 1.18 (t, *J* = 7.0, 3H); $^{13}$C NMR (126 MHz, DMSO-$d_6$) δ 172.5, 161.8, 154.4, 146.2, 130.6, 125.0, 122.3, 115.2, 67.2, 60.0, 30.1, 24.1, 14.1; ESI-HR-MS calcd for $(C_{19}H_{20}N_2O_5+H)^+$ 357.1450, found 357.1447.

**Ethyl 4-{4-[4-(7-heptanamido-1,8-naphthyridin-2-ylcarbamoyl)phenyl]diazenyl}phenoxy)-butanoate (5)**

To a stirred solution of **4** (38 mg, 0.140 mmol), DMAP (5 mg, 0.035 mmol), and **3** (100 mg, 0.280 mmol) in 12 mL of $CH_2Cl_2$ cooled to 0 °C in an ice bath, was added EDC (54 mg, 0.280 mmol), and the mixture was warmed to ambient temperature over several hours and stirred for 24 h. The solution was diluted with 20 mL of $CH_2Cl_2$ and was washed with water (3 × 20 mL), a saturated aqueous solution of sodium bicarbonate (2 × 20 mL), and brine (20 mL). The organic layer was dried over sodium sulfate, concentrated in vacuo and purified by silica gel column chromatography with MeOH–$CH_2Cl_2$ (5:95 *v*/*v*, $R_f$ = 0.65) to afford **5** (61 mg, 71%) as an orange solid. mp 228-229 °C; $^1$H NMR (500 MHz, $CDCl_3$) δ 9.14 (s, 1H), 8.61 (s, 1H), 8.59 (d, *J* = 8.5, 1H), 8.46 (d, *J* = 9.0, 1H), 8.18 (d, *J* = 8.5, 1H), 8.14 (d, *J* = 9.0, 1H), 8.05 (d, *J* = 8.5, 2H), 7.93 (d, *J* = 9.0, 2H), 7.92 (d, *J* = 8.5, 2H), 7.00 (d, *J* = 9.0, 2H), 4.16 (q, *J* = 7.5, 2H), 4.10 (t, *J* = 6.0, 2H), 2.54 (t, *J* = 7.0, 2H), 2.44 (t, *J* = 7.5, 2H), 2.13-2.18 (m, 2H), 1.68-1.74 (m, 2H), 1.34 (t, *J* = 7.0, 3H), 1.26-1.28 (m, 6H), 0.86 (t, *J* = 7.0, 3H); $^{13}$C NMR (126 MHz, $CDCl_3$) δ 173.2, 172.6, 165.4, 162.2, 155.2, 154.2, 154.1, 153.8, 147.1, 139.3, 139.2, 134.8, 128.5, 125.4, 123.0, 118.6, 114.9, 113.8, 113.7, 67.2, 60.7, 38.1, 31.6, 30.8, 28.9, 25.3, 24.6, 22.6, 14.4, 14.1; ESI-HR-MS calcd for $(C_{34}H_{38}N_6O_5+H)^+$ 611.2982, found 611.2984.

**4-(4-{[4-(*tert*-Butoxycarbonyl)phenyl]diazenyl}phenoxy)butanoic acid (6)**

A suspension of **2** (0.148 g, 0.359 mmol) in 12 mL of a 1:1 (*v*/*v*) mixture of 10% aqueous LiOH and ethanol was stirred at room temperature for 1 h. The mixture was neutralized by dropwise addition of a 5% aqueous solution of HCl, concentrated in vacuo to remove ethanol, and extracted with $CH_2Cl_2$ (3 × 50 mL). The combined organic layers were washed with saturated aqueous sodium bicarbonate (20 mL), and brine (2 × 20 mL). The organic layer was dried over

sodium sulfate, concentrated in vacuo and purified by silica gel column chromatography MeOH–$CH_2Cl_2$ (5:95 *v*/*v*, $R_f$ = 0.33) to afford 121 mg (88%) of **6** as an orange solid: mp 202-203 °C; $^1$H NMR (500 MHz, $CDCl_3$) δ 8.11 (d, *J* = 8.0, 2H), 7.94 (d, *J* = 9.0, 2H), 7.88 (d, *J* =8.5, 2H), 7.01 (d, *J* = 9.0, 2H), 4.13 (t, *J* = 6.5, 2H), 2.63 (t, *J* = 7.0, 2H), 2.15-2.21 (m, 2H), 1.62 (s, 9H); $^{13}$C NMR (126 MHz, $CDCl_3$) δ 178.7, 165.5, 161.8, 155.2, 147.2, 133.3, 130.5, 125.3, 122.4, 114.9, 81.5, 67.0, 30.5, 28.3, 24.4; ESI-HR-MS calcd for $(C_{21}H_{24}N_2O_5+H)^+$ 385.1763, found 385.1765.

***tert*-Butyl 4-({4-[4-(7-isobutyramido-1,8-naphthyridin-2-ylamino)-4-oxobutoxy]phenyl}diazenyl)benzoate (8)**

To a stirred solution of **7** (30 mg, 0.130 mmol), DMAP (4 mg, 0.033 mmol), and **6** (50 mg, 0.130 mmol) in 10 mL of $CH_2Cl_2$ cooled to 0 °C in an ice bath, was added EDC (27 mg, 0.143 mmol), and the reaction mixture was warmed to ambient temperature over several hours and stirred for 24 h. The solution was diluted with 20 mL of $CH_2Cl_2$ and washed with water (2 × 20 mL), a saturated solution of aqueous sodium bicarbonate (20 mL), and brine (20 mL). The organic layer was dried over sodium sulfate, concentrated in vacuo and purified by silica gel column chromatography with MeOH–$CH_2Cl_2$ (1:9 *v*/*v*, $R_f$ = 0.72) to afford 47 mg (60%) of **8** as an orange solid: mp 208-209 °C; $^1$H NMR (500 MHz, $CDCl_3$) δ 8.86 (bs, 1H), 8.51 (bs, 1H), 8.46 (d, *J* = 8.5, 1H), 8.43 (d, *J* = 9.0, 1H), 8.13 (d, *J* = 9.0, 1H), 8.12 (d, *J* = 8.0, 1H), 8.10 (d, *J* = 8.0, 2H), 7.90 (d, *J* = 8.5, 2H), 7.86 (d, *J* = 8.5, 2H), 6.97 (d, *J* = 8.5, 2H), 4.13 (t, *J* = 6.0, 2H), 2.71 (t, *J* = 7.0, 2H), 2.60-2.64 (m, 1H), 2.23-2.27 (m, 2H), 1.61 (s, 9H), 1.26 (d, *J* = 7.0, 6H); $^{13}$C NMR (126 MHz, $CDCl_3$) δ 176.2, 171.7, 165.4, 161.8, 155.1, 154.2, 154.0, 147.2, 139.3, 139.2, 133.3, 130.6, 130.5, 125.3, 122.3, 118.5, 114.9, 113.7, 113.6, 81.5, 67.2, 37.0, 34.1, 28.3, 24.6, 19.4; ESI-HR-MS calcd for $(C_{33}H_{36}N_6O_5+H)^+$ 597.2825, found 597.2825.

***tert*-Butyl 4-[(4-{4-[7-(2-ethylhexanamido)-1,8-naphthyridin-2-ylamino]-4-oxobutoxy}phenyl)diazenyl]benzoate (10)**

To a stirred solution of **9** (31 mg, 0.108 mmol), DMAP (4 mg, 0.033 mmol) and **6** (42 mg, 0.108 mmol) in 10 mL $CH_2Cl_2$ cooled to 0 °C in an ice bath, was added EDC (25 mg, 0.119 mmol) and the mixture was warmed to ambient temperature over several hours and stirred for 24 h. The solution was diluted with 20 mL $CH_2Cl_2$ and was washed with water (2 × 20 mL), a saturated aqueous solution of sodium bicarbonate (20 mL), and brine (20 mL). The organic layer was dried over sodium sulfate, concentrated in vacuo and purified by silica gel column

chromatography with MeOH–$CH_2Cl_2$ (5:95 *v*/*v*, $R_f$ =0.41) to afford 47 mg (62%) of **10** as an orange solid: mp 198-199 °C; $^1$H NMR (500 MHz, $CDCl_3$) δ 8.88 (bs, 1H), 8.50 (bs, 1H), 8.48 (d, *J* = 9.0, 1H), 8.43 (d, *J* = 9.0, 1H), 8.14 (d, *J* = 9.0, 1H), 8.13 (d, *J* = 8.5, 1H), 8.10 (d, *J* = 8.0, 2H), 7.90 (d, *J* = 9.0, 2H), 7.87 (d, *J* = 8.5, 2H), 6.97 (d, *J* = 9.0, 2H), 4.13 (t, *J* = 6.0, 2H), 2.71 (t, *J* = 7.0, 2H), 2.23-2.27 (m, 2H), 2.17-2.21 (m, 1H), 1.67-1.74 (m, 2H), 1.61 (s, 9H), 1.48-1.55 (m, 2H), 1.25-1.30 (m, 2H), 0.93 (t, *J* = 7.5, 3H), 0.84 (t, *J* = 7.0, 3H); $^{13}$C NMR (126 MHz, $CDCl_3$) δ 175.6, 171.7, 165.4, 161.8, 155.1, 154.1, 153.8, 147.2, 139.3, 139.2, 133.3, 130.6, 125.3, 124.3, 122.3, 119.3, 118.5, 114.9, 114.3, 113.9, 113.6, 81.5, 67.2, 60.0, 34.1, 32.5, 29.9, 28.3, 26.1, 24.6, 22.8, 14.0, 12.1; ESI-HR-MS calcd for $(C_{37}H_{44}N_6O_5+H)^+$ 653.3451, found 653.3453.

**_tert_-Butyl 4-[(4-{2-[2-(3-butylureido)-7-(cyclohexylmethyl)-4-oxo-4,7-dihydro-3*H*-pyrrolo-[2,3-*d*]pyrimidin-6-yl]acetoxy}phenyl)diazenyl]benzoate (12)**

To a stirred solution of **11** (36 mg, 0.089 mmol), DMAP (3 mg, 0.025 mmol) and **1** (19 mg, 0.064 mmol) in 10 mL of $CH_2Cl_2$ cooled to 0 °C in an ice bath, was added EDC (22 mg, 0.116 mmol) and the mixture was warmed to ambient temperature over several hours and stirred for 24 h. The solution was diluted with 20 mL $CH_2Cl_2$ and was washed with water (2 × 20 mL), a saturated aqueous solution of sodium bicarbonate (20 mL), and brine (20 mL). The organic layer was dried over sodium sulfate, concentrated in vacuo and purified by silica gel column chromatography with MeOH–$CH_2Cl_2$ (5:95 *v*/*v*, $R_f$ = 0.24) to afford 47 mg (35%) of **12** as an orange-yellow solid: mp 150-151 °C; $^1$H NMR (500 MHz, $CDCl_3$) δ 9.65 (bs, 1H), 9.36 (bs, 1H), 8.94 (bs, 1H), 8.13 (d, *J* = 8.5, 2H), 7.98 (d, *J* = 9.0, 1H), 7.91 (d, *J* = 8.5, 2H), 7.65 (bs, 1H), 7.31 (d, *J* = 9.0, 2H), 4.03 (s, 2H), 3.93 (d, *J* = 7.0, 2H), 3.45-3.49 (m, 2H), 1.80-1.84 (m, 2H), 1.75-1.77 (m, 1H), 1.63-1.68 (m, 4H), 1.62 (s, 9H), 1.46-1.51 (m, 2H), 1.14-1.19 (m, 4H), 0.99 (t, *J* = 7.5, 3H), 0.84-0.91 (m, 2H); $^{13}$C NMR (126 MHz, $CDCl_3$) δ 180.9, 168.1, 165.3, 154.8, 153.8, 153.0, 151.8, 149.2, 146.4, 134.0, 132.2, 130.5, 125.5, 124.5, 122.7, 122.3, 120.1, 116.1, 104.0, 81.7, 50.5, 49.5, 40.4, 40.3, 39.1, 38.9, 33.4, 32.3, 32.1, 30.9, 29.8, 28.3, 26.4, 25.8, 20.4, 14.0; ESI-HR-MS calcd for $(C_{37}H_{45}N_7O_6+H)^+$ 684.3510, found 684.3508.

**4-[(4-Hydroxyphenyl)diazenyl]benzoic acid (13)**

To a suspension of 4-aminobenzoic acid (1.24 g, 9.00 mmol) in 25 mL of a 2.0 M aqueous solution of hydrochloric acid cooled to 0 °C in an ice bath was added dropwise a solution of

sodium nitrite (1.04 g, 15.1 mmol) in water (5.0 mL). The pale yellow mixture was stirred at 0 °C for 20 min and added slowly to an ice-cold solution of phenol (706 mg, 7.50 mmol), sodium hydroxide (260 mg, 6.50 mmol) and potassium carbonate (1.40 g, 10.1 mmol) in 20 mL of water. The mixture was stirred for 1 h while being cooled in an ice-water bath and then warmed to ambient temperature and stirred for 3 h. The brown suspension was neutralized with a 2 M aqueous solution of HCl and filtered. The solid was dissolved in MeOH and dry loaded on silica gel and purified by column chromatography with MeOH-$CH_2Cl_2$ (5:95 *v*/*v*, $R_f$ = 0.32) to afford 1.18 g, (65%) of **13** as an orange solid: mp 274-275 °C; $^1$H NMR (500 MHz, $CD_3OD$) δ 8.15 (d, *J* = 8.5, 2H), 7.88 (d, *J* = 8.5, 2H), 7.85 (d, *J* = 9.0, 2H), 6.92 (d, *J* = 9.0, 2H); $^{13}$C NMR (126 MHz, $CD_3OD$) δ 169.2, 162.9, 156.8, 147.6, 133.0, 131.8, 126.4, 123.2, 116.8; ESI-HR-MS calcd for $(C_{13}H_{10}N_2O_3+H)^+$ 243.0770, found 243.0772.

**4-{[4-(5-Bromopentyloxy)phenyl]diazenyl}benzoic acid (14)**

A mixture of **13** (120 mg, 0.500 mmol), 1,5-dibromopentane (137 mg, 0.596 mmol), potassium carbonate (207 mg, 1.50 mmol), and potassium iodide (9 mg, 0.0542 mmol) in 15 mL anhydrous ethanol was stirred and heated under reflux for 20 h. The mixture was cooled to ambient temperature and concentrated in vacuo. The residue was dissolved in 50 mL of $CH_2Cl_2$ and washed with 30 mL of a 2 M aqueous solution of HCl and 30 mL of brine. The organic layer was concentrated in vacuo and purified by silica gel column chromatography with MeOH-$CH_2Cl_2$ (1:9 *v*/*v*, $R_f$ = 0.43) to afford 120 mg (62%) of **14** as an orange solid: mp 216-217 °C; $^1$H NMR (500 MHz, DMSO-$d_6$) δ 13.22 (bs, 1H), 8.12 (d, *J* = 8.5, 2H), 7.93 (d, *J* = 9.0, 2H), 7.91 (d, *J* = 8.0, 2H), 7.15 (d, *J* = 9.0, 2H), 4.11 (t, *J* =6.5, 2H), 3.58 (t, *J* = 7.0, 2H), 1.86-1.92 (m, 2H), 1.76-1.82 (m, 2H), 1.53-1.59 (m, 2H); $^{13}$C NMR (126 MHz, DMSO-$d_6$) δ 166.8, 162.0, 154.4, 146.1, 132.2, 130.6, 125.0, 122.2, 115.2, 67.9, 35.1, 31.9, 27.7, 24.2; ESI-HR-MS calcd for $(C_{18}H_{19}N_2O_3Br+H)^+$ 391.0657, found 391.0657.

**Ethyl 4-{[4-(5-bromopentyloxy)phenyl]diazenyl}benzoate (15)**

To a stirred solution of **14** (125 mg, 0.320 mmol), DMAP (12 mg, 0.098 mmol), and anhydrous ethanol (2.76 g, 60 mmol) in 12 mL of $CH_2Cl_2$ cooled to 0 °C in an ice bath, was added EDC (67 mg, 0.350 mmol). The mixture was warmed to ambient temperature over several hours, stirred for 24 h, and concentrated in vacuo. The residue was dissolved in 40 mL of $CH_2Cl_2$ and washed with water (2 × 20 mL), a saturated aqueous solution of sodium bicarbonate (2 × 20 mL),

and with brine (20 mL). The organic layer was dried over sodium sulfate, concentrated in vacuo and purified by silica gel column chromatography with MeOH-$CH_2Cl_2$ (1:99 *v*/*v*, $R_f$ = 0.83) to afford **15** (102 mg, 76%) as an orange solid: mp 98-99 °C; $^1$H NMR (500 MHz, $CDCl_3$) δ 8.17 (d, *J* = 8.5, 2H), 7.94 (d, *J* = 8.5, 2H), 7.90 (d, *J* = 8.0, 2H), 7.00 (d, *J* = 8.5, 2H), 4.41 (q, *J* = 7.0, 2H), 4.06 (t, *J* = 6.0, 2H), 3.45 (t, *J* = 6.5, 2H), 1.93-1.98 (m, 2H), 1.83-1.88 (m, 2H), 1.62-1.69 (m, 2H), 1.42 (t, *J* = 7.0, 3H); $^{13}$C NMR (126 MHz, $CDCl_3$) δ 166.3, 162.2, 155.4, 147.0, 131.6, 130.6, 125.3, 122.4, 114.9, 68.1, 61.3, 33.7, 32.5, 28.5, 24.9, 14.5; ESI-HR-MS calcd for $(C_{20}H_{23}N_2O_3Br+H)^+$ 419.0970, found 419.0969.

**Ethyl 4-{[4-(5-azidopentyloxy)phenyl]diazenyl}benzoate (16)**

A mixture of **15** (100 mg, 0.238 mmol), sodium azide (372 mg, 0.572 mmol), tetrabutyl amonium bromide (100 mg, 0.31 mmol) and five drops of acetonitrile in 15 mL of $CH_2Cl_2$ was stirred and heated under reflux for 4 h. The mixture was cooled to ambient temperature and concentrated in vacuo. The residue was taken up in 60 mL of $CH_2Cl_2$, washed with brine (3 × 30 mL), and concentrated in vacuo to afford 131 mg of crude **16** as an orange solid, which was used for next step without further purification. The product was confirmed with HR-ESI-MS calcd for $(C_{20}H_{23}N_5O_3+H)^+$ 382.1879, found 382.1879.

**Ethyl 4-[(4-{5-[4-(3-{2-[2-(3-butylureido)-7-(cyclohexylmethyl)-4-oxo-4,7-dihydro-3*H*-pyrrolo[2,3-*d*]pyrimidin-6-yl]acetoxy}propyl)-1*H*-1,2,3-triazol-1-yl]pentyloxy}phenyl)dia-zenyl]benzoate (18)**

To a stirred solution of **16** (131 mg) and **17** (40 mg, 0.085 mmol) in 15 mL of $CH_2Cl_2$ was added a solution of copper(II)sulfate pentahydrate (10 mg, 0.04 mmol) and sodium ascorbate (36 mg, 0.182 mmol) in 2 mL water. The mixture was stirred at room temperature for 24 h. The water layer was separated and washed with 40 mL of $CH_2Cl_2$. The combined $CH_2Cl_2$ layers were washed with brine (2 × 20 mL), dried over sodium sulfate, concentrated in vacuo and purified by silica gel column chromatography with MeOH-$CH_2Cl_2$ (1:9 *v*/*v*, $R_f$ = 0.46) to afford 52 mg (71%) of **18** as an orange yellow solid: mp 159-160°C; $^1$H NMR (500 MHz, $CDCl_3$) δ 11.24 (bs, 1H), 9.39 (bs, 1H), 8.97 (bs, 1H), 8.15 (d, *J* = 8.0, 2H), 7.89 (d, *J* = 9.0, 2H), 7.87 (d, *J* = 9.0, 2H), 7.39 (bs, 1H), 6.94 (d, *J* = 9.0, 2H), 6.85 (bs, 1H), 4.40 (q, *J* = 7.0, 2H), 4.37 (t, *J* = 6.0, 2H), 4.23 (t, *J* = 7.5, 2H), 4.15 (t, *J* = 7.0, 2H), 3.99 (t, *J* = 7.5, 2H), 3.82 (d, *J* = 7.0, 2H), 3.73 (s, 2H), 2.25-2.29 (m, 2H), 2.03-2.09 (m, 2H), 2.00-2.04 (m, 1H), 1.96-2.05 (m, 2H), 1.80-1.83 (m, 2H),

1.84-1.86 (m, 2H), 1.69-1.72 (m, 4H), 1.62-1.66 (m, 4H), 1.54-1.58 (m, 2H), 1.46-1.52 (m, 4H), 1.41 (t, $J$ = 9.0, 3H), 0.97 (t, $J$ = 7.0, 3H); $^{13}$C NMR (126 MHz, $CDCl_3$) δ 170.0, 166.3, 162.1, 159.3, 155.4, 154.1, 148.6, 147.0, 146.4, 131.6, 130.6, 127.5, 126.3, 125.3, 122.4, 114.8, 103.5, 103.3, 102.5, 83.1, 69.4, 67.9, 64.4, 64.0, 61.3, 50.3, 49.3, 40.2, 39.0, 33.2, 32.2, 31.2, 30.1, 28.6, 28.2, 27.4, 26.3, 25.9, 23.2, 22.0, 20.5, 15.3, 14.5, 14.0; ESI-HR-MS calcd for $(C_{45}H_{58}N_{10}O_7+H)^+$ 851.4568, found 851.4562.

## $^{1}$H and $^{13}$C NMR spectra

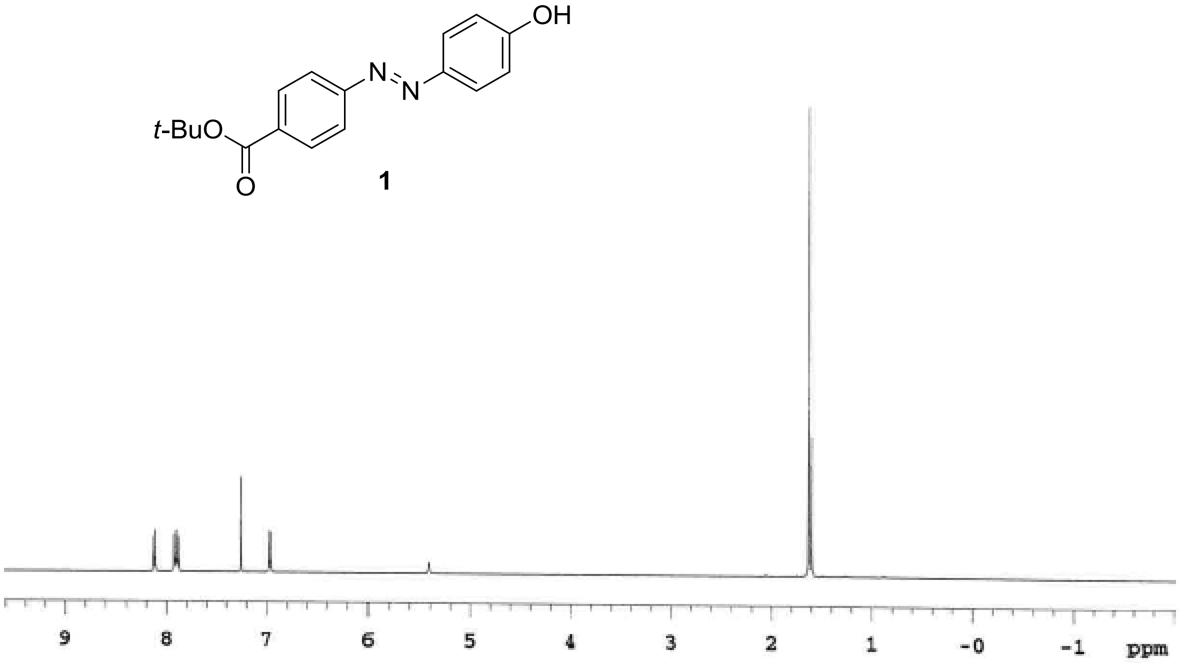


**Figure S1:** Compound **1**, $^{1}$H NMR (500 MHz, $CDCl_3$).

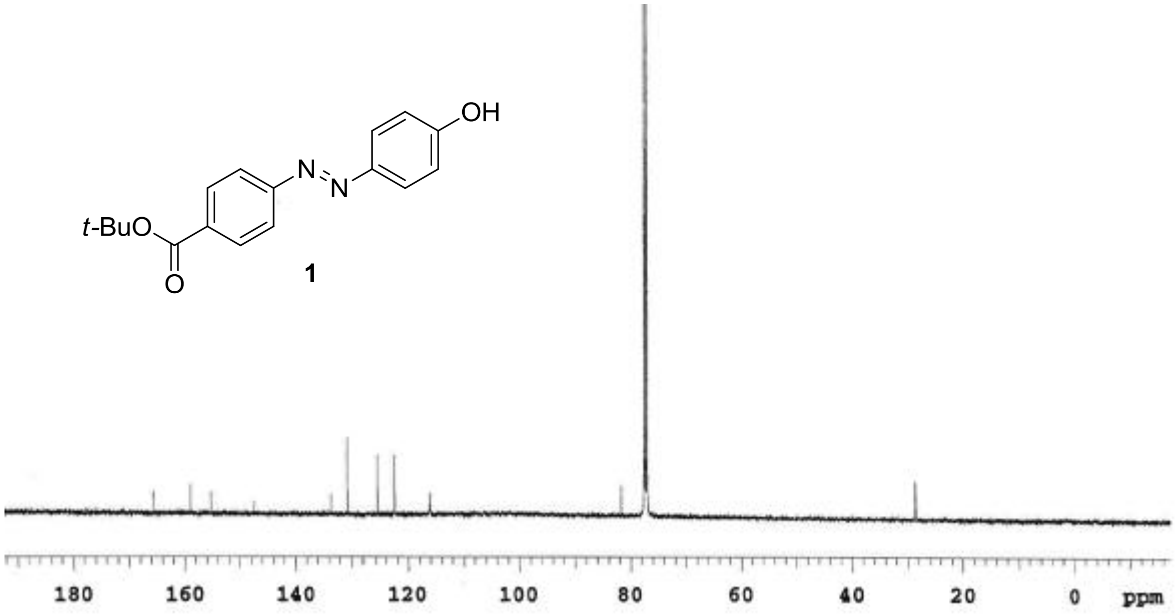


**Figure S2:** Compound **1**, $^{13}$C NMR (126 MHz, $CDCl_3$).

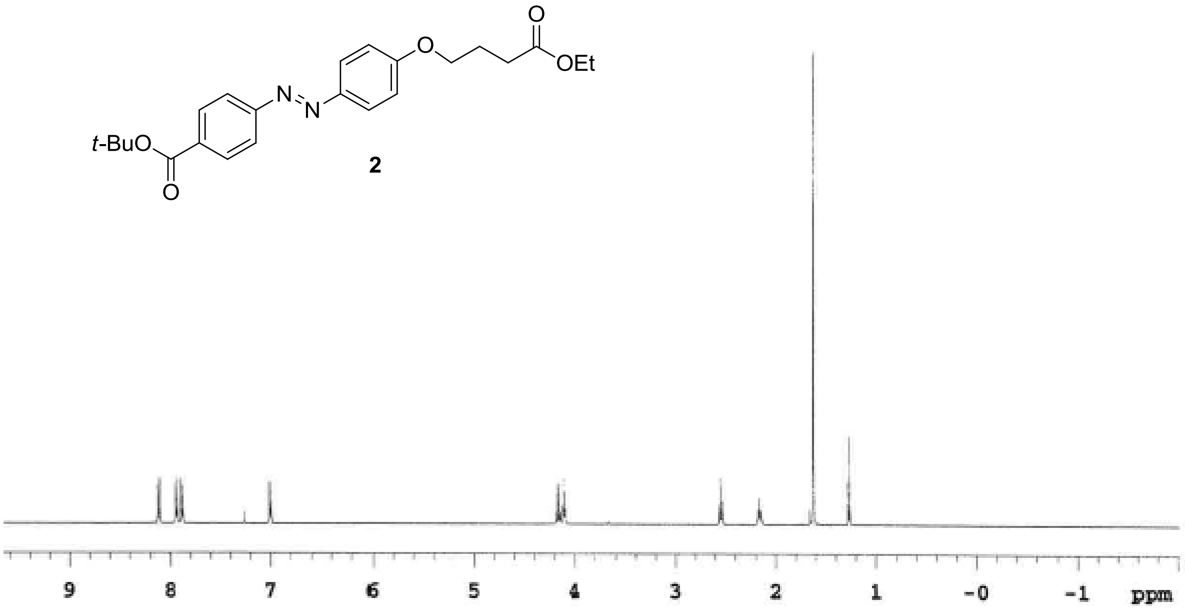


**Figure S3:** Compound **2**, $^{1}$H NMR (500 MHz, $CDCl_3$).

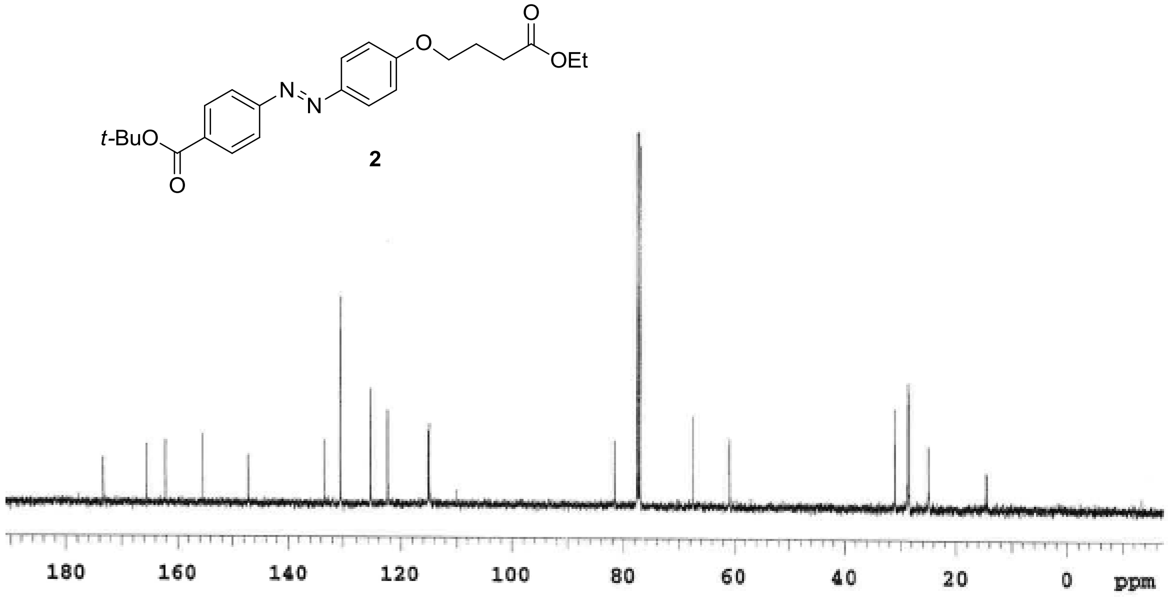


**Figure S4:** Compound **2**, $^{13}$C NMR (126 MHz, $CDCl_3$).

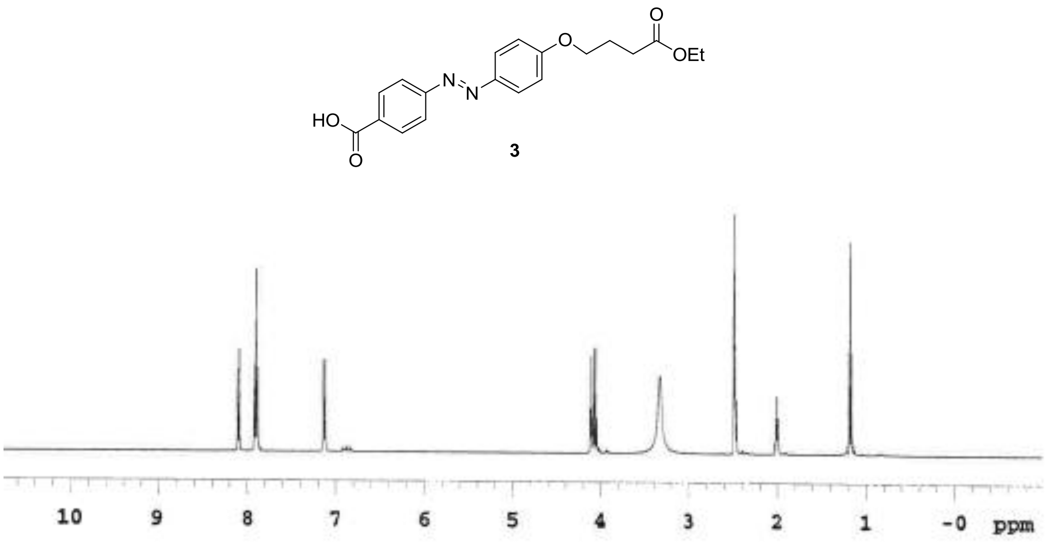


**Figure S5:** Compound **3**, $^{1}$H NMR (500 MHz, DMSO-$d_6$).

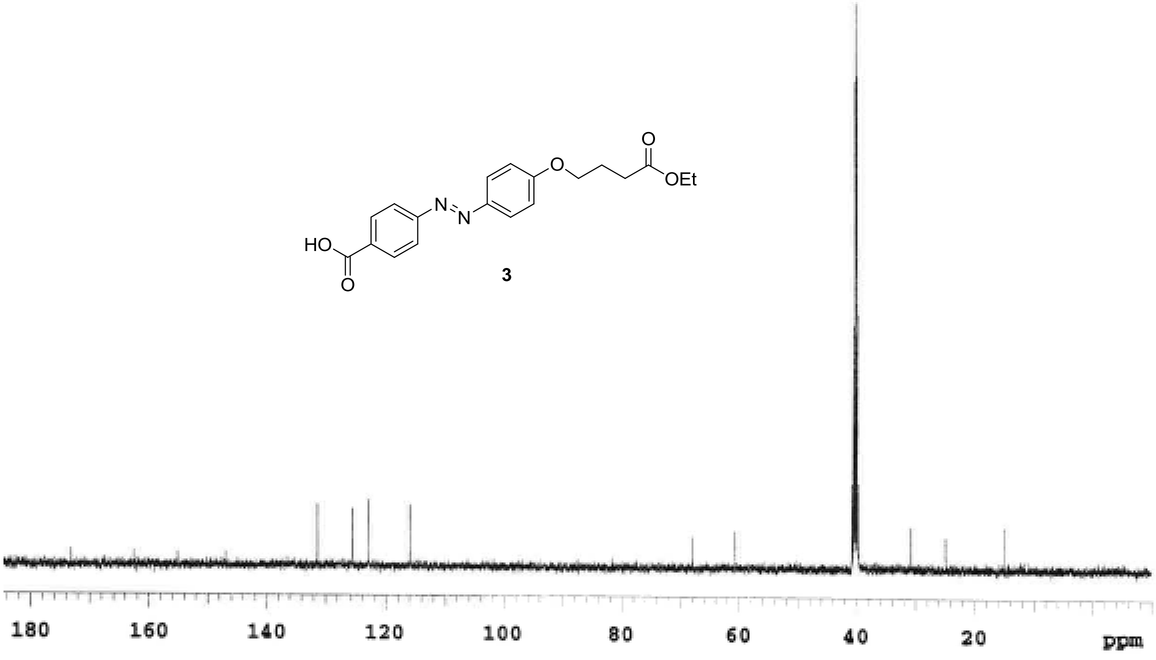


**Figure S6:** Compound **3**, $^{13}$C NMR (126 MHz, DMSO-$d_6$).

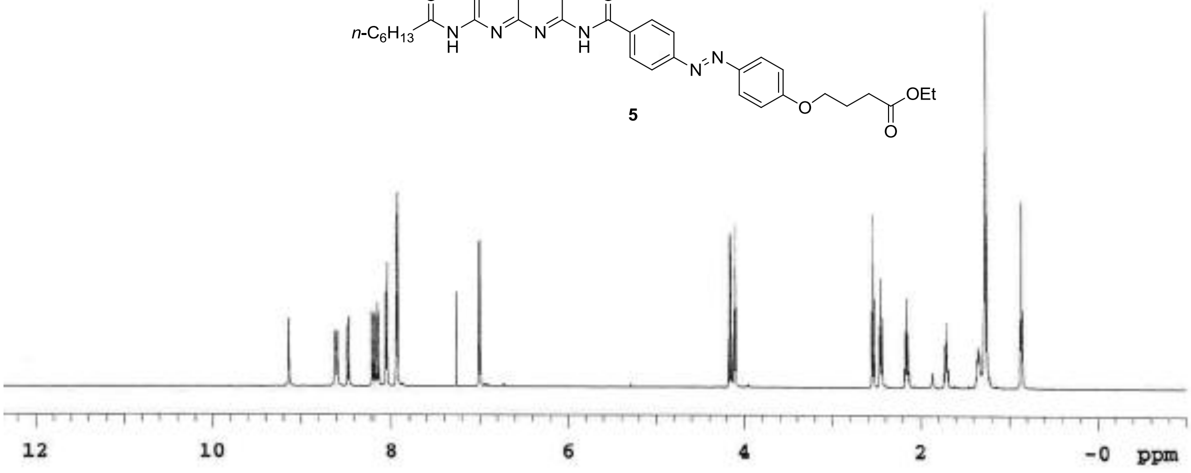


**Figure S7:** Compound **5**, $^{1}$H NMR (500 MHz, $CDCl_3$).

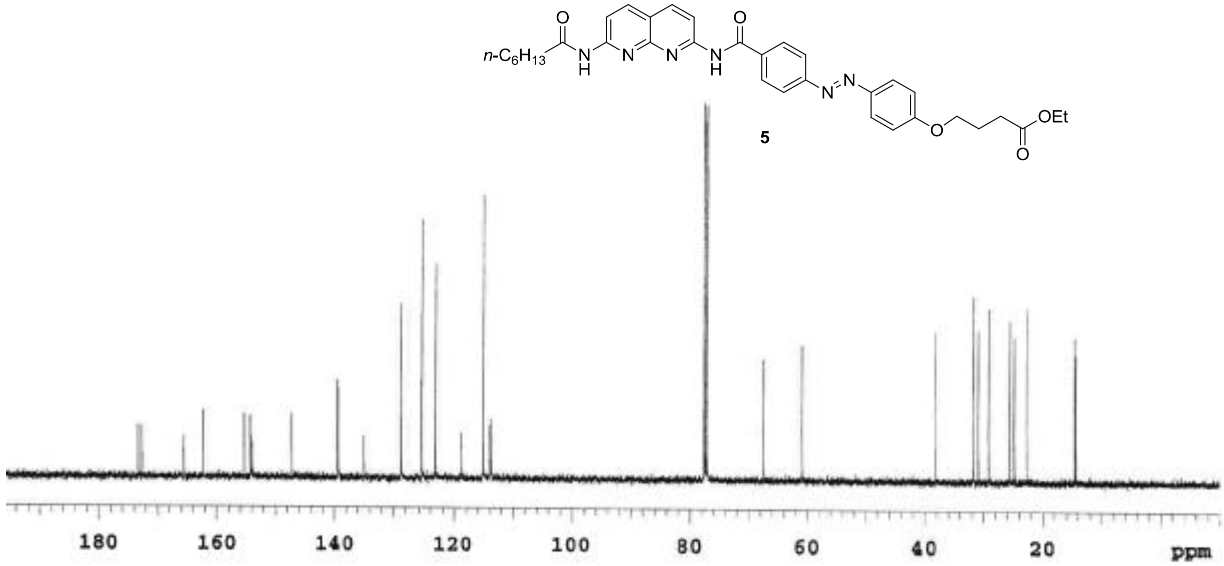


**Figure S8:** Compound **5**, $^{13}$C NMR (126 MHz, $CDCl_3$).

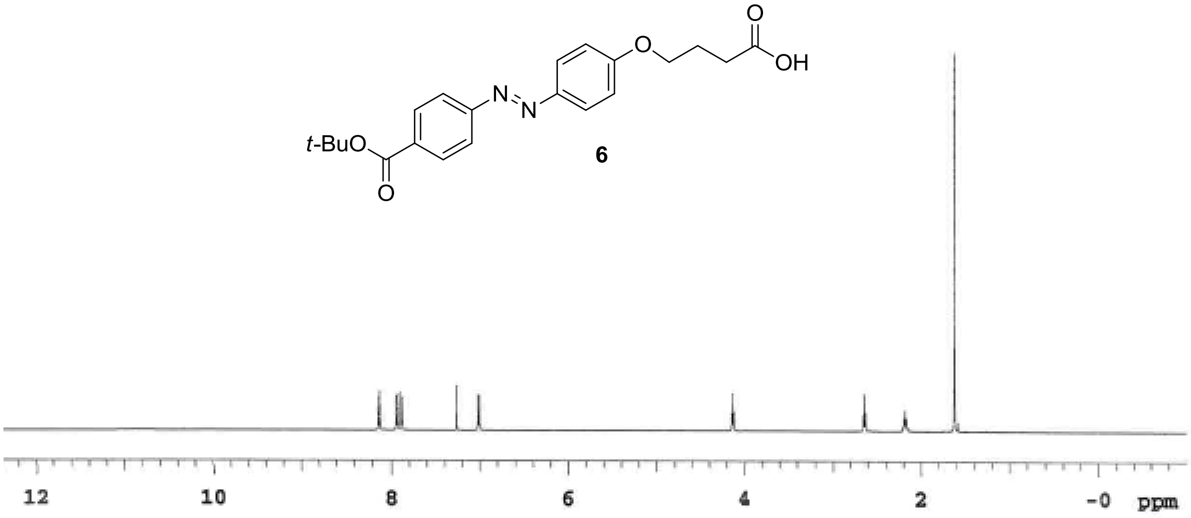


**Figure S9:** Compound **6**, $^{1}$H NMR (500 MHz, $CDCl_3$).

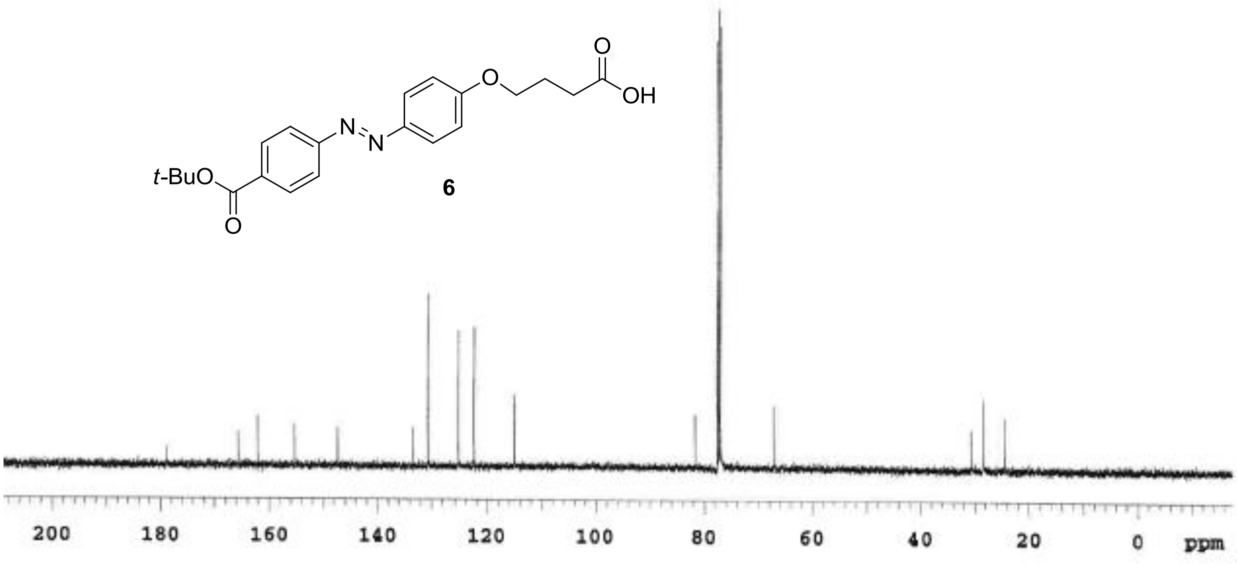


**Figure S10:** Compound **6**, $^{13}$C NMR (126 MHz, $CDCl_3$).

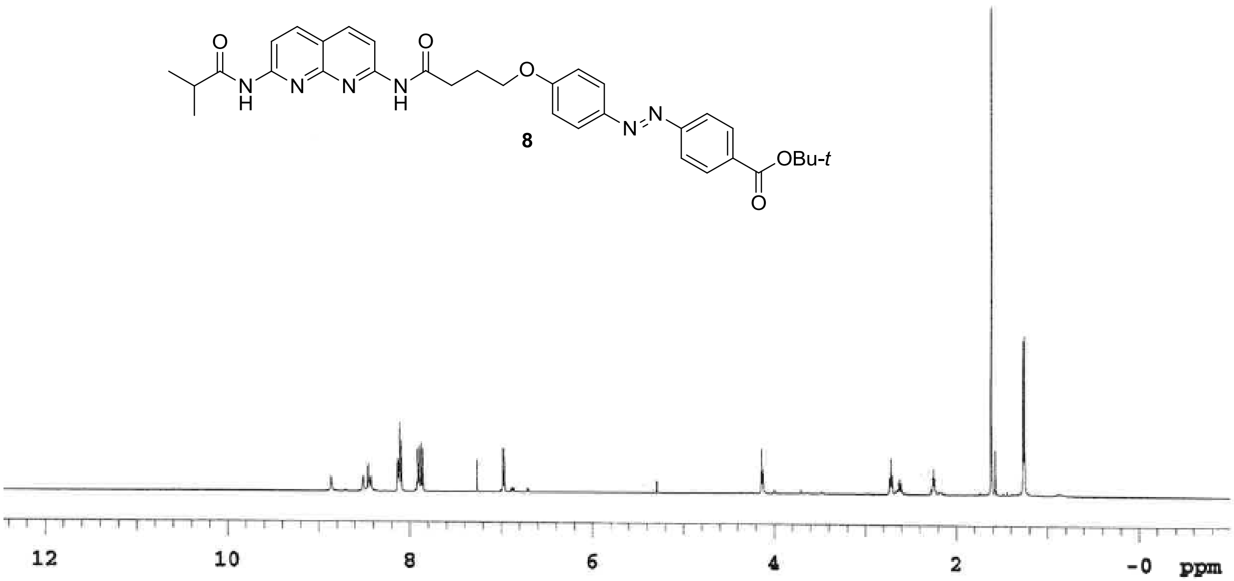


**Figure S11:** Compound **8**, $^{1}$H NMR (500 MHz, $CDCl_3$).

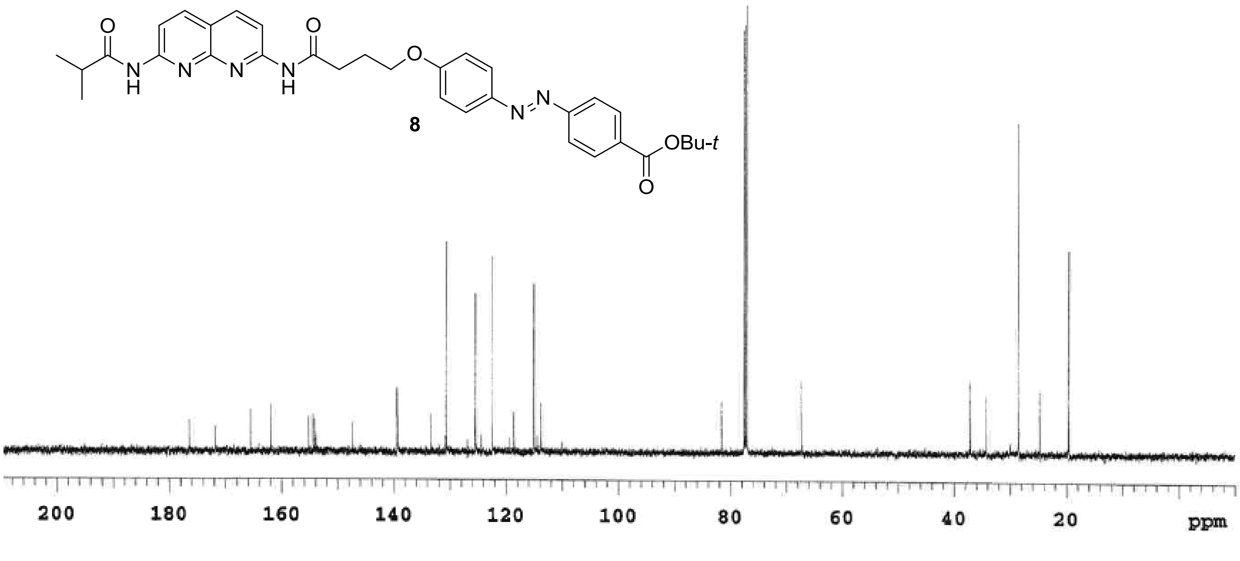


**Figure S12:** Compound **8**, $^{13}$C NMR (126 MHz, $CDCl_3$).

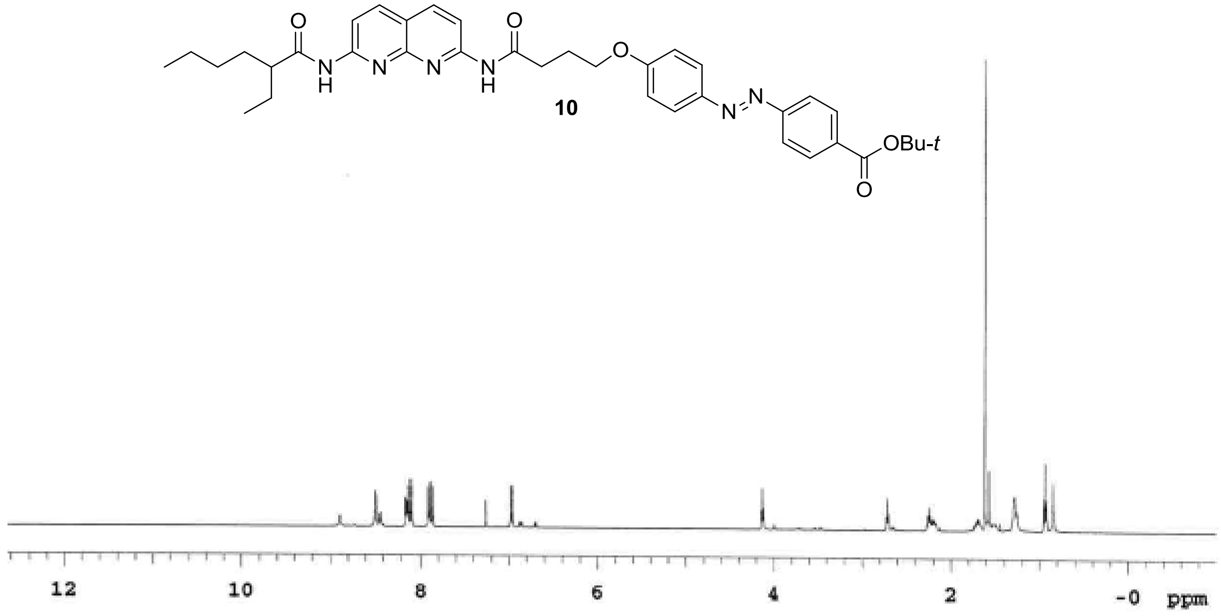


**Figure S13:** Compound **10**, $^{1}$H NMR (500 MHz, CDCl$_3$).

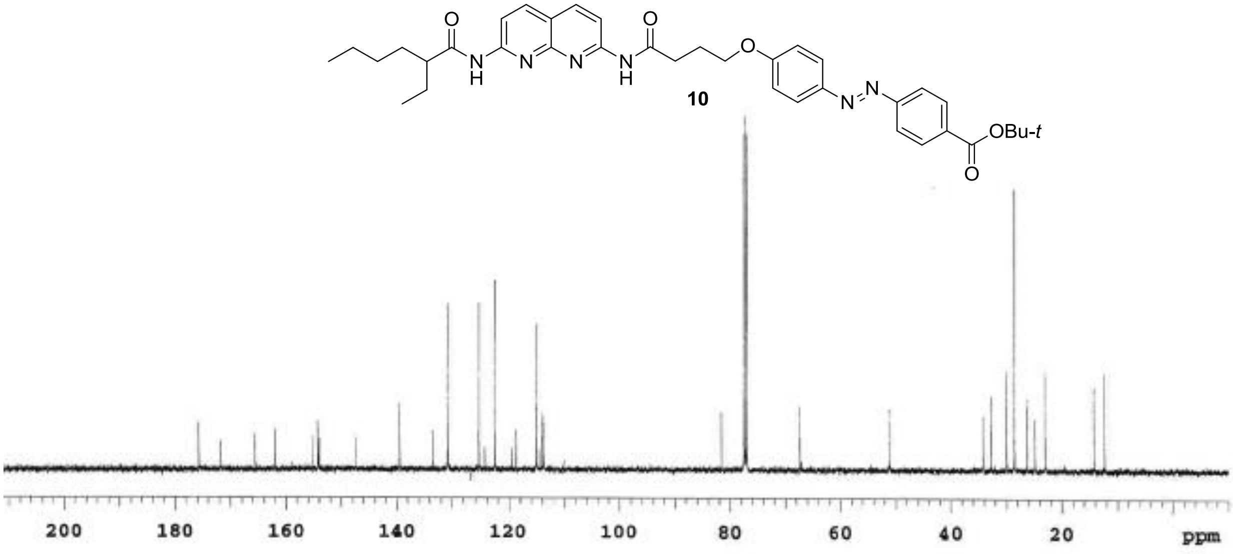


**Figure S14:** Compound **10**, $^{13}$C NMR (126 MHz, CDCl$_3$).

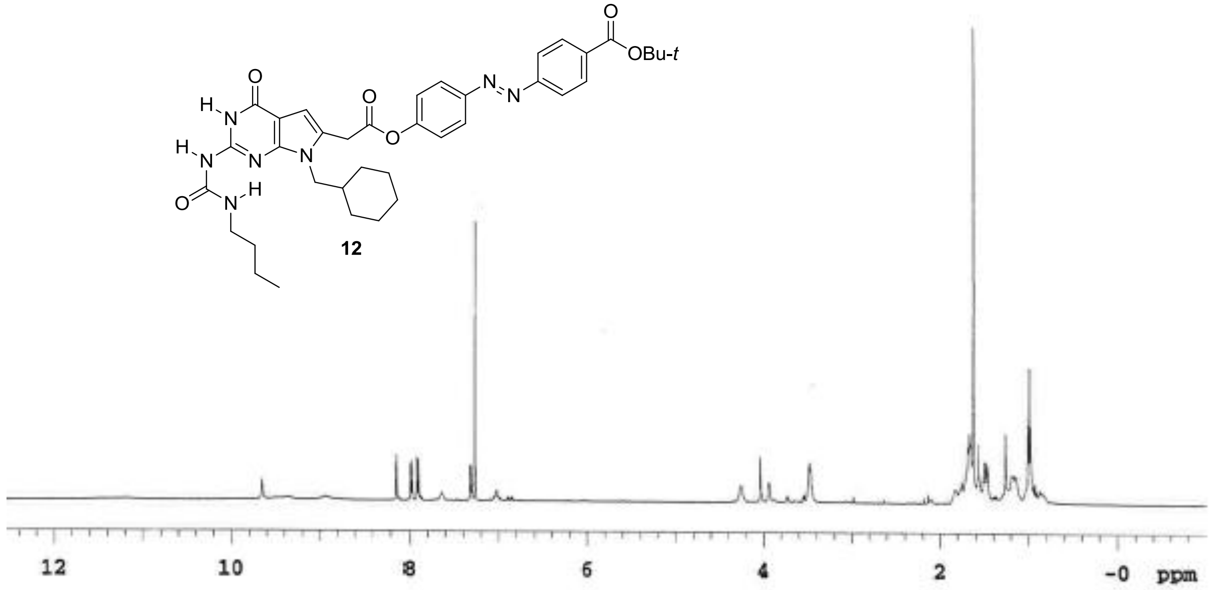


**Figure S15:** Compound **12**, $^{1}$H NMR (500 MHz, $CDCl_3$).

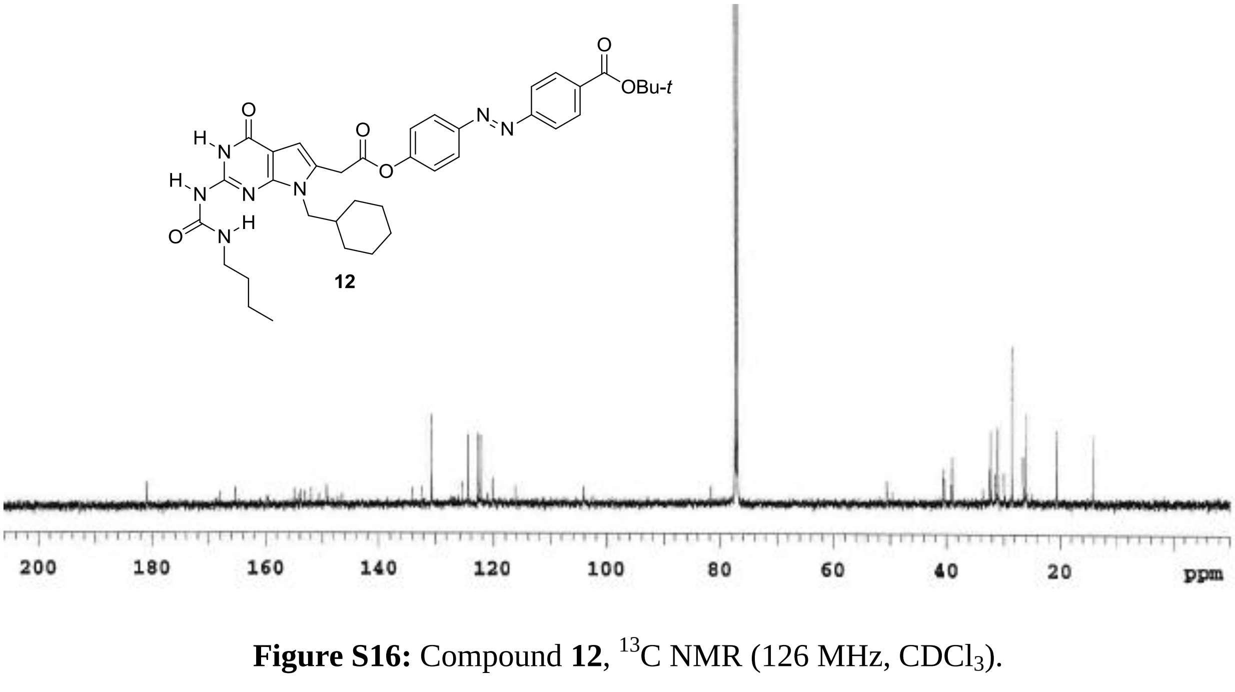


**Figure S16:** Compound **12**, $^{13}$C NMR (126 MHz, $CDCl_3$).

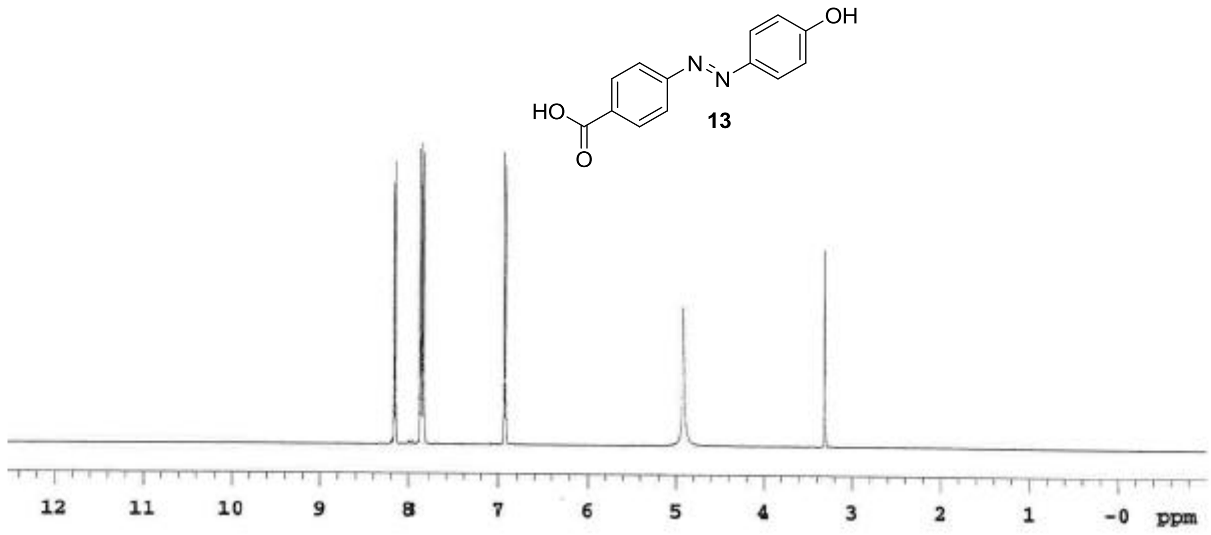


**Figure S17:** Compound **13**, $^{1}$H NMR (500 MHz, $CD_3OD$).

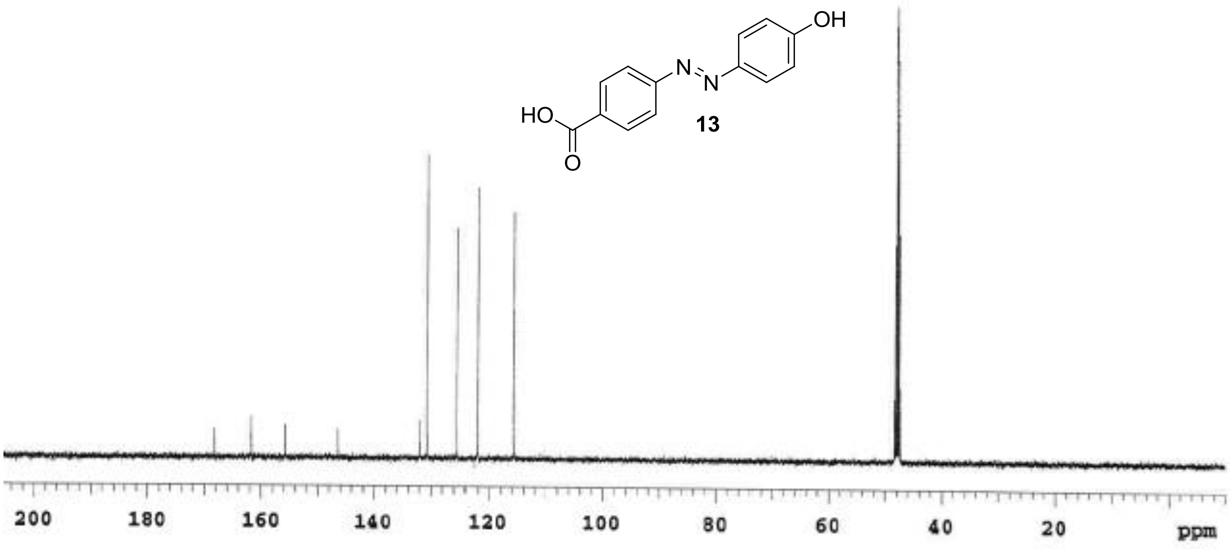


**Figure S18:** Compound **13**, $^{13}$C NMR (126 MHz, $CD_3OD$).

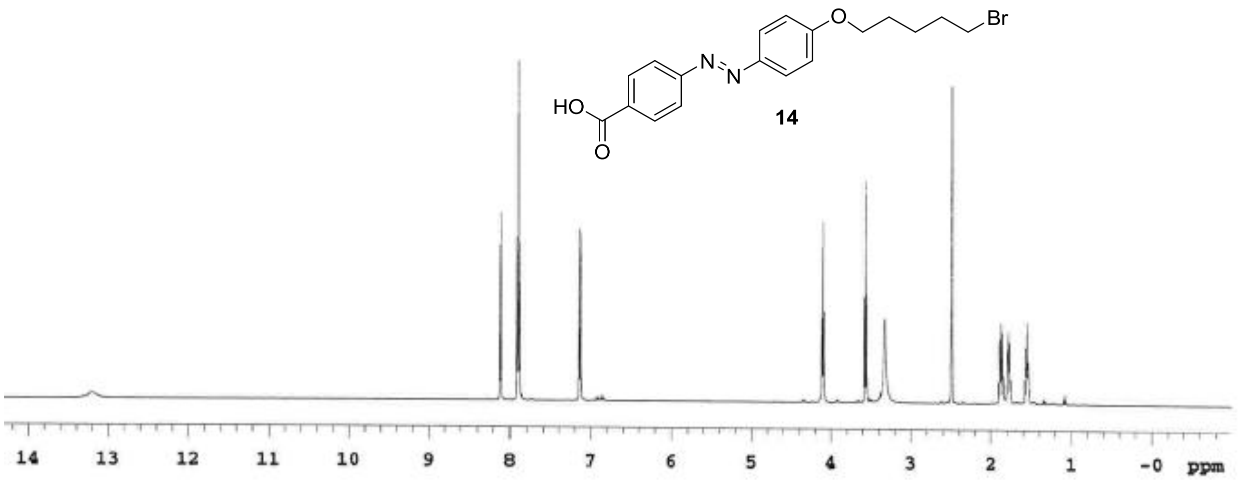


**Figure S19:** Compound **14**, $^{1}$H NMR (500 MHz, DMSO-$d_6$).

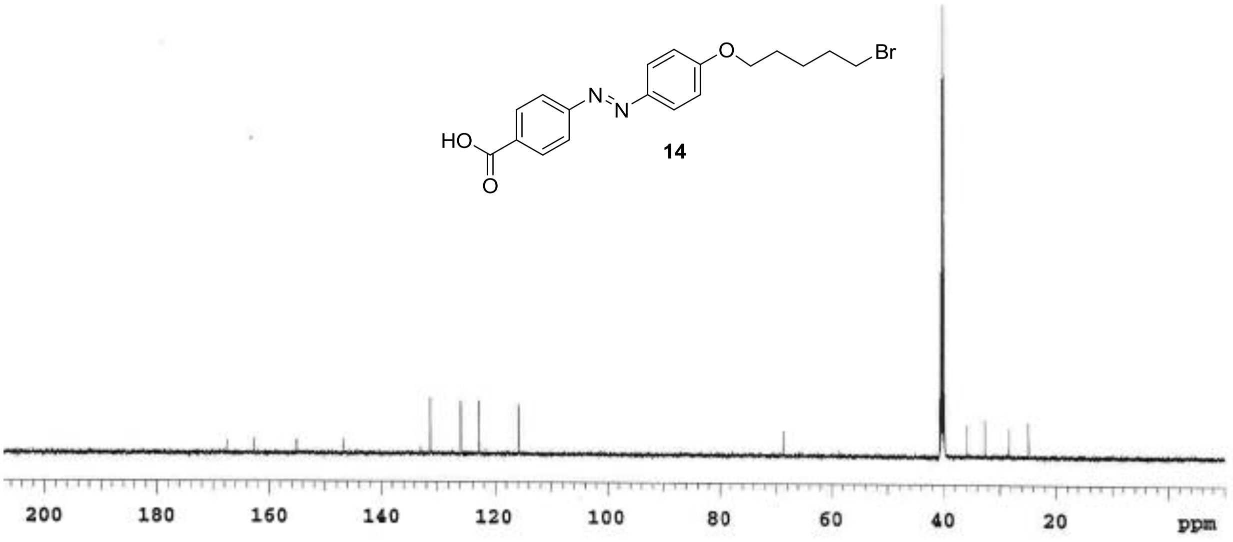


**Figure S20:** Compound **14**, $^{13}$C NMR (126 MHz, DMSO-$d_6$).

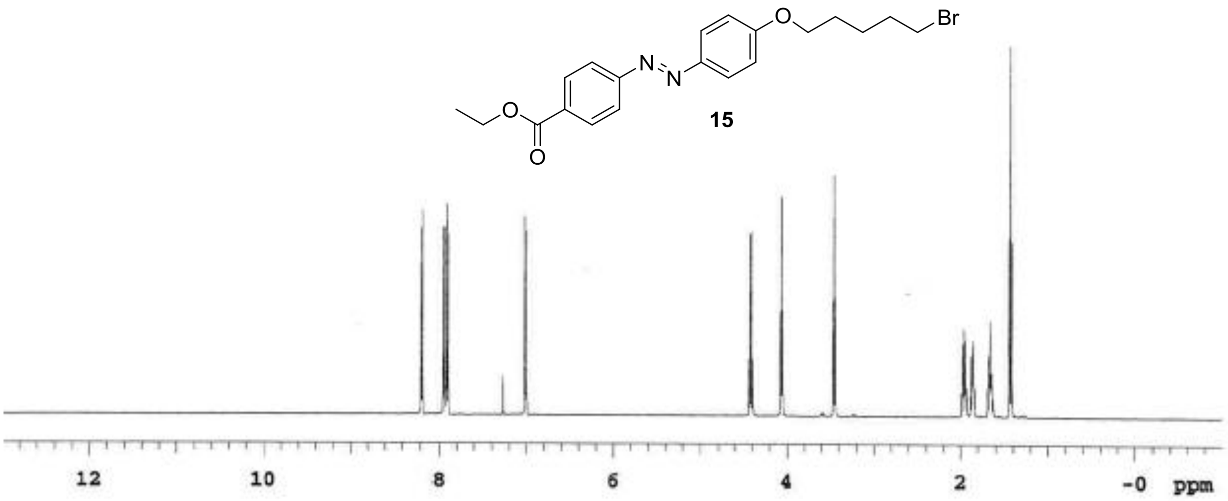


**Figure S21:** Compound **15**, $^{1}$H NMR (500 MHz, $CDCl_3$).

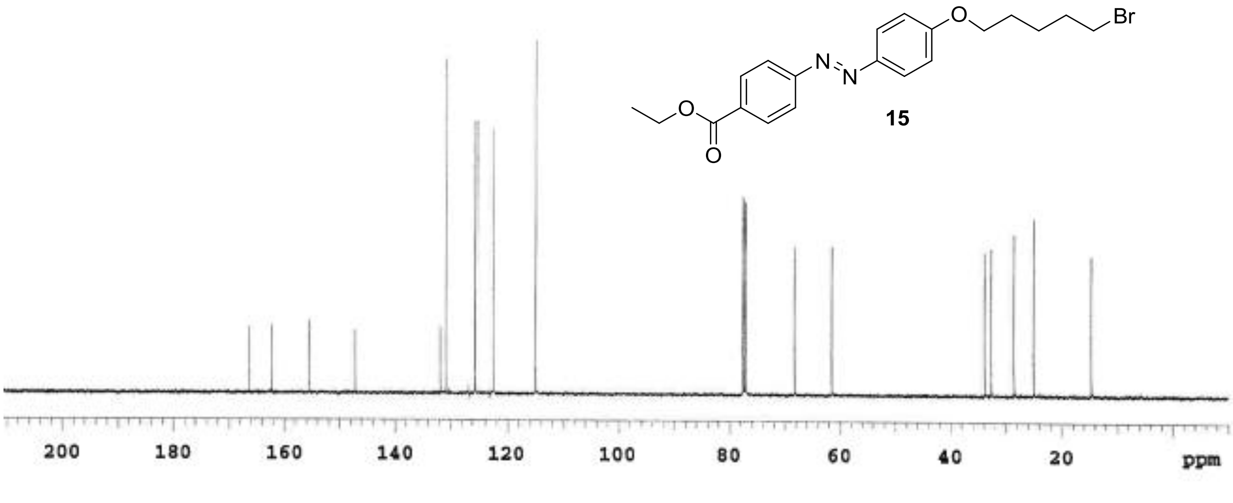


**Figure S22:** Compound **15**, $^{13}$C NMR (126 MHz, $CDCl_3$).

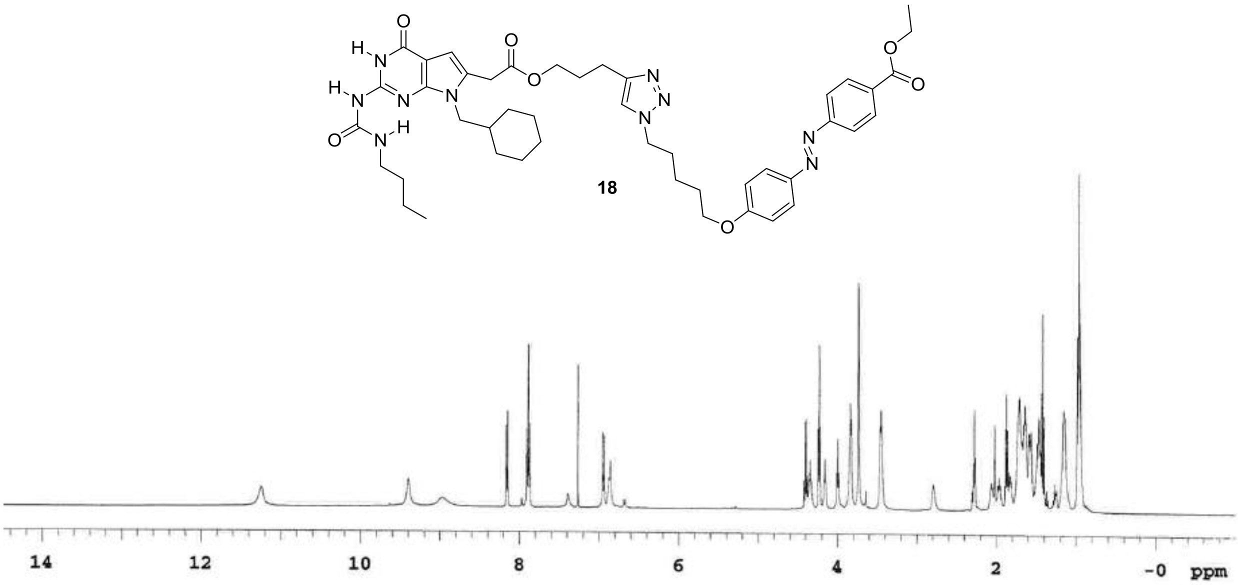


**Figure S23:** Compound **18**, $^{1}$H NMR (500 MHz, $CDCl_3$).

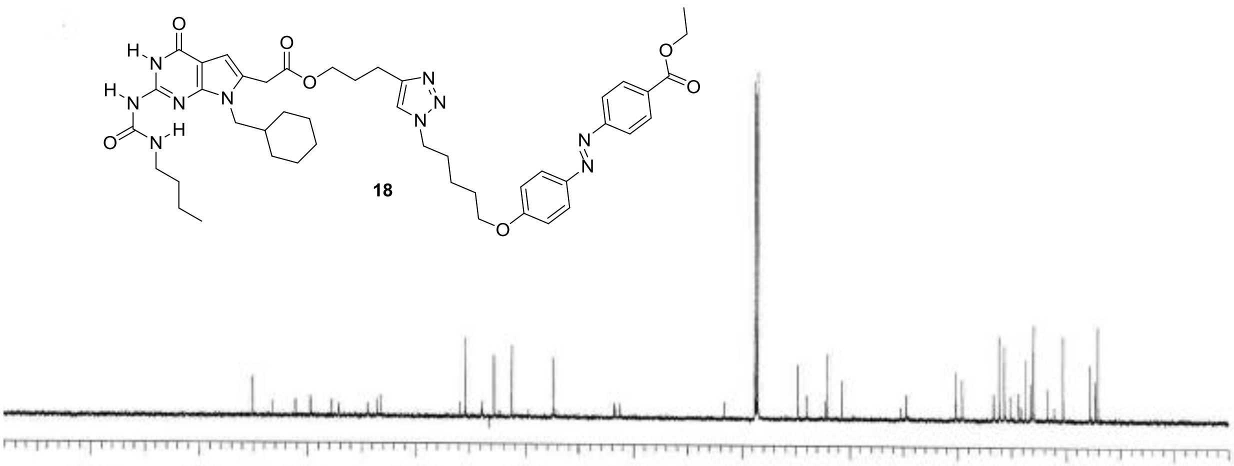


**Figure S24:** Compound **18**, $^{13}$C NMR (126 MHz, $CDCl_3$).

# Experimental composition reports of HR-ESI-MS

**Elemental Composition Report** Page 1

**Single Mass Analysis**
Tolerance = 10.0 PPM / DBE: min = -1.5, max = 150.0
Element prediction: Off
Number of isotope peaks used for i-FIT = 3

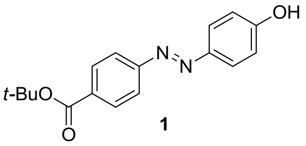


Monoisotopic Mass, Even Electron Ions
265 formula(e) evaluated with 3 results within limits (all results (up to 1000) for each mass)
Elements Used:
C: 0-100 H: 0-150 N: 0-5 O: 0-5 Na: 0-1
Zhang, Yagang, scz-zyg-3-3 University of Ilinois, SCS, Mass Spectrometry Lab Q-tof UE521
Qtof_35844 45 (3.221) AM (Cen,3, 80.00, Ar,15000.0,716.46,0.70,LS 3); Sm (SG, 2x5.00); Cm (45:46) 1: TOF MS ES+ 1.88e+003

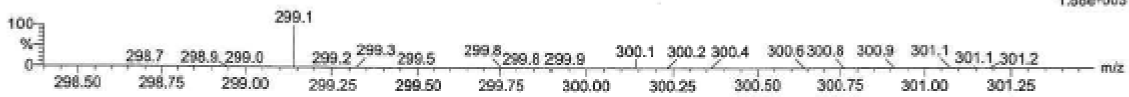


| | | | | | | |
|---|---|---|---|---|---|---|
| Minimum: | | | | -1.5 | | |
| Maximum: | | 5.0 | 10.0 | 150.0 | | |

| Mass | Calc. Mass | mDa | PPM | DBE | i-FIT | Formula |
|---|---|---|---|---|---|---|
| 299.1395 | 299.1396 | -0.1 | -0.3 | 9.5 | 1.0 | C17 H19 N2 O3 |
| | 299.1412 | -1.7 | -5.7 | 10.5 | 0.8 | C20 H20 O Na |
| | 299.1372 | 2.3 | 7.7 | 6.5 | 6.8 | C15 H20 N2 O3 Na |

**Figure S25:** Elemental composition report of compound **1** (HR-ESI-MS).

**Elemental Composition Report** Page 1

**Single Mass Analysis**
Tolerance = 10.0 PPM / DBE: min = -1.5, max = 150.0
Element prediction: Off
Number of isotope peaks used for i-FIT = 3

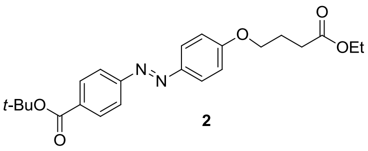


Monoisotopic Mass, Even Electron Ions
363 formula(e) evaluated with 4 results within limits (all results (up to 1000) for each mass)
Elements Used:
C: 0-100 H: 0-150 N: 0-5 O: 0-5 Na: 0-1
Zhang, Yagang, scz-zyg-3-5 University of Ilinois, SCS, Mass Spectrometry Lab Q-tof UE521
Qtof_35845 31 (2.221) AM (Cen,3, 80.00, Ar,15000.0,716.46,0.70,LS 3); Sm (SG, 2x5.00); Cm (27:31) 1: TOF MS ES+ 1.84e+003

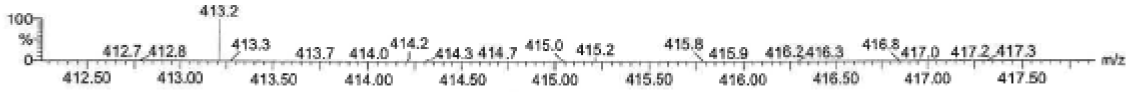


| | | | | | | |
|---|---|---|---|---|---|---|
| Minimum: | | | | -1.5 | | |
| Maximum: | | 5.0 | 10.0 | 150.0 | | |

| Mass | Calc. Mass | mDa | PPM | DBE | i-FIT | Formula |
|---|---|---|---|---|---|---|
| 413.2079 | 413.2076 | 0.3 | 0.7 | 10.5 | 26.9 | C23 H29 N2 O5 |
| | 413.2093 | -1.4 | -3.4 | 11.5 | 29.2 | C26 H30 O3 Na |
| | 413.2052 | 2.7 | 6.5 | 7.5 | 31.6 | C21 H30 N2 O5 Na |
| | 413.2117 | -3.8 | -9.2 | 14.5 | 30.8 | C28 H29 O3 |

**Figure S26:** Elemental composition report of compound **2** (HR-ESI-MS).

**Elemental Composition Report** **Page 1**

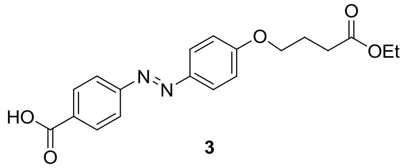


**Single Mass Analysis**
Tolerance = 10.0 PPM / DBE: min = -1.5, max = 150.0
Element prediction: Off
Number of isotope peaks used for i-FIT = 3

Monoisotopic Mass, Even Electron Ions
40 formula(e) evaluated with 1 results within limits (all results (up to 1000) for each mass)
Elements Used:
C: 0-100 H: 0-150 N: 1-3 O: 4-6
Zhang, Yagang, scz-zyg-3-10 University of Ilnois, SCS, Mass Spectrometry Lab Q-tof UE521
Qtof_36005 51 (3.650) AM (Cen,3, 80.00, Ar,15000.0,716.46,0.70,LS 3); Sm (SG, 2x3.00); Cm (51:53) 1: TOF MS ES+ 2.47e+003

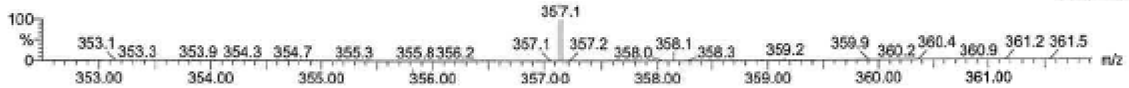


| | | | | | | |
|---|---|---|---|---|---|---|
| Minimum: | | | | -1.5 | | |
| Maximum: | | 5.0 | 10.0 | 150.0 | | |
| Mass | Calc. Mass | mDa | PPM | DBE | i-FIT | Formula |
| 357.1447 | 357.1450 | -0.3 | -0.8 | 10.5 | 12.6 | C19 H21 N2 O5 |

**Figure S27:** Elemental composition report of compound **3** (HR-ESI-MS).

**Elemental Composition Report** **Page 1**

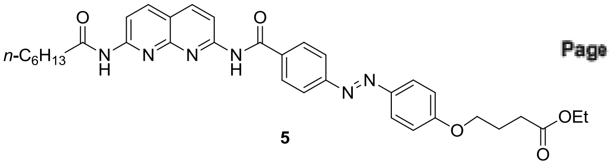


**Single Mass Analysis**
Tolerance = 10.0 PPM / DBE: min = -1.5, max = 150.0
Element prediction: Off
Number of isotope peaks used for i-FIT = 3

Monoisotopic Mass, Even Electron Ions
170 formula(e) evaluated with 3 results within limits (all results (up to 1000) for each mass)
Elements Used:
C: 0-100 H: 0-150 N: 5-6 O: 0-5 Na: 0-1
Zhang, Yagang, scz-zyg-3-9 University of Ilnois, SCS, Mass Spectrometry Lab Q-tof UE521
Qtof_35959 30 (2.150) AM (Cen,3, 80.00, Ar,15000.0,716.46,0.70,LS 3); Sm (SG, 2x3.00); Cm (30) 1: TOF MS ES+ 1.29e+003

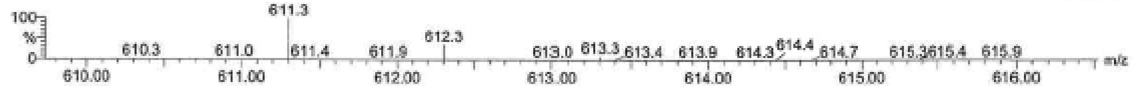


| | | | | | | |
|---|---|---|---|---|---|---|
| Minimum: | | | | -1.5 | | |
| Maximum: | | 5.0 | 10.0 | 150.0 | | |
| Mass | Calc. Mass | mDa | PPM | DBE | i-FIT | Formula |
| 611.2984 | 611.2982 | 0.2 | 0.3 | 18.5 | 1.5 | C34 H39 N6 O5 |
| | 611.2958 | 2.6 | 4.3 | 15.5 | 0.4 | C32 H40 N6 O5 Na |
| | 611.2923 | 6.1 | 10.0 | 27.5 | 15.7 | C41 H35 N6 |

**Figure S28:** Elemental composition report of compound **5** (HR-ESI-MS).

**Elemental Composition Report** **Page 1**

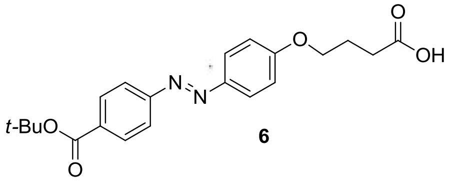


6

**Single Mass Analysis**
Tolerance = 10.0 PPM / DBE: min = -1.5, max = 150.0
Element prediction: Off
Number of isotope peaks used for i-FIT = 3

Monoisotopic Mass, Even Electron Ions
133 formula(e) evaluated with 1 results within limits (all results (up to 1000) for each mass)
Elements Used:
C: 0-150 H: 0-200 N: 2-6 O: 2-7
Zhang, Yagang, 3-17-2 University of Illinois, SCS, Mass Spectrometry Lab Q-tof UE521
Qtof_36207 26 (1.864) AM (Cen,3, 80.00, Ar,15000.0,734.47,0.70,LS 3); Sm (SG, 2x5.00); Cm (23:26) 1: TOF MS ES+ 9.28e+002

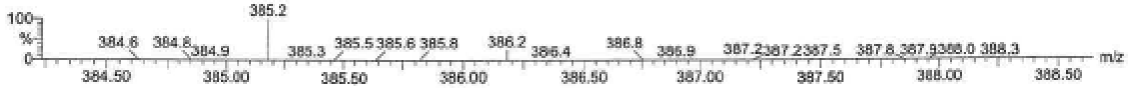


| | | | | | | |
|---|---|---|---|---|---|---|
| Minimum: | | | | -1.5 | | |
| Maximum: | | 5.0 | 10.0 | 150.0 | | |
| Mass | Calc. Mass | mDa | PPM | DBE | i-FIT | Formula |
| 385.1765 | 385.1763 | 0.2 | 0.5 | 10.5 | 0.7 | C21 H25 N2 O5 |

**Figure S29:** Elemental composition report of compound **6** (HR-ESI-MS).

**Elemental Composition Report** **Page 1**

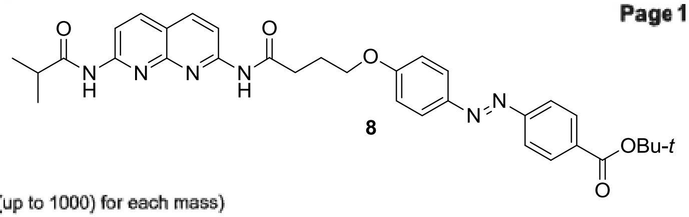


8

**Single Mass Analysis**
Tolerance = 10.0 PPM / DBE: min = -1.5, max = 150.0
Element prediction: Off
Number of isotope peaks used for i-FIT = 3

Monoisotopic Mass, Even Electron Ions
357 formula(e) evaluated with 4 results within limits (all results (up to 1000) for each mass)
Elements Used:
C: 0-150 H: 0-200 N: 0-6 O: 0-6
Zhang, Yagang, scz-zyg-3-21 University of Illinois, SCS, Mass Spectrometry Lab Q-tof UE521
Qtof_36535 30 (2.150) AM (Cen,3, 80.00, Ar,15000.0,734.47,0.70,LS 3); Sm (SG, 2x3.00); Cm (29:30) 1: TOF MS ES+ 2.95e+003

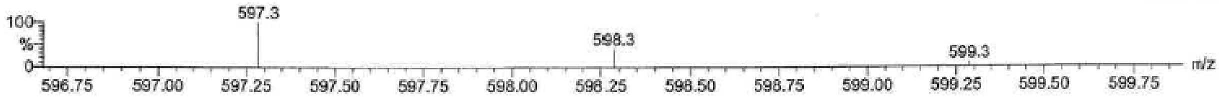


| | | | | | | |
|---|---|---|---|---|---|---|
| Minimum: | | | | -1.5 | | |
| Maximum: | | 5.0 | 10.0 | 150.0 | | |
| Mass | Calc. Mass | mDa | PPM | DBE | i-FIT | Formula |
| 597.2825 | 597.2825 | 0.0 | 0.0 | 18.5 | 0.1 | C33 H37 N6 O5 |
| | 597.2794 | 3.1 | 5.2 | 26.5 | 34.7 | C44 H37 O2 |
| | 597.2866 | -4.1 | -6.9 | 22.5 | 8.1 | C38 H37 N4 O3 |
| | 597.2767 | 5.8 | 9.7 | 27.5 | 18.0 | C40 H33 N6 |

**Figure S30:** Elemental composition report of compound **8** (HR-ESI-MS).

**Elemental Composition Report** **Page 1**

**Single Mass Analysis**
Tolerance = 10.0 PPM / DBE: min = -1.5, max = 150.0
Element prediction: Off
Number of isotope peaks used for i-FIT = 3

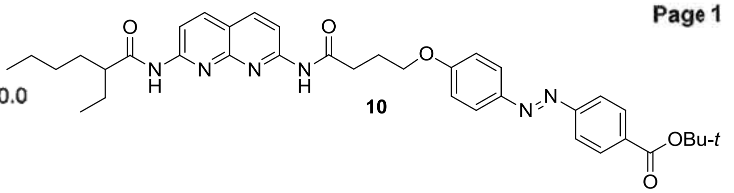


Monoisotopic Mass, Even Electron Ions
441 formula(e) evaluated with 5 results within limits (all results (up to 1000) for each mass)
Elements Used:
C: 0-150 H: 0-200 N: 0-6 O: 0-7
Zhang, Yagang, scz-zyg-3-20 University of Illinois, SCS, Mass Spectrometry Lab Q-tof UE521
Qtof_36452 21 (1.507) AM (Cen,3, 80.00, Ar,15000.0,734.47,0.70,LS 3); Sm (SG, 2x3.00); Cm (20:21) 1: TOF MS ES+ 1.63e+003

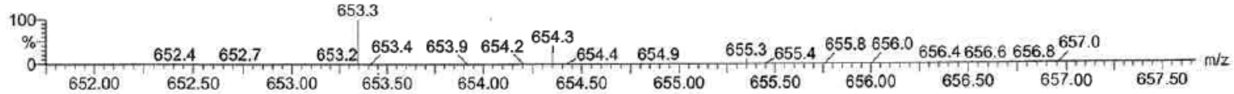


| | | | | | | |
|---|---|---|---|---|---|---|
| Minimum: | | | | -1.5 | | |
| Maximum: | | 5.0 | 10.0 | 150.0 | | |

| Mass | Calc. Mass | mDa | PPM | DBE | i-FIT | Formula |
|---|---|---|---|---|---|---|
| 653.3453 | 653.3451 | 0.2 | 0.3 | 18.5 | 0.9 | C37 H45 N6 O5 |
| | 653.3478 | -2.5 | -3.8 | 17.5 | 4.7 | C41 H49 O7 |
| | 653.3420 | 3.3 | 5.1 | 26.5 | 25.4 | C48 H45 O2 |
| | 653.3492 | -3.9 | -6.0 | 22.5 | 8.5 | C42 H45 N4 O3 |
| | 653.3393 | 6.0 | 9.2 | 27.5 | 15.5 | C44 H41 N6 |

**Figure S31:** Elemental composition report of compound **10** (HR-ESI-MS).

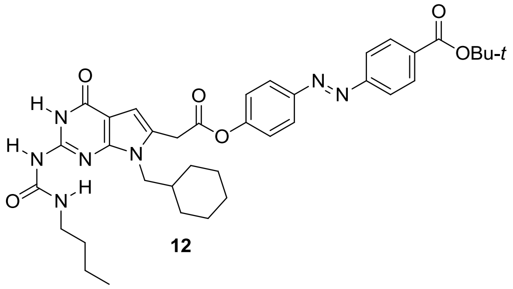


**Elemental Composition Report** **Page 1**

**Single Mass Analysis**
Tolerance = 10.0 PPM / DBE: min = -1.5, max = 150.0
Element prediction: Off
Number of isotope peaks used for i-FIT = 3

Monoisotopic Mass, Even Electron Ions
135 formula(e) evaluated with 3 results within limits (all results (up to 1000) for each mass)
Elements Used:
C: 0-150 H: 0-200 N: 5-7 O: 4-6 S: 0-1
Zhang, Yagang, scz-zyg-3-15-2 University of Illinois, SCS, Mass Spectrometry Lab Q-tof UE521
Qtof_36134A 53 (3.793) AM (Cen,3, 80.00, Ar,15000.0,734.47,0.70,LS 3); Sm (SG, 2x3.00); Cm (53:54) 1: TOF MS ES+ 3.08e+003

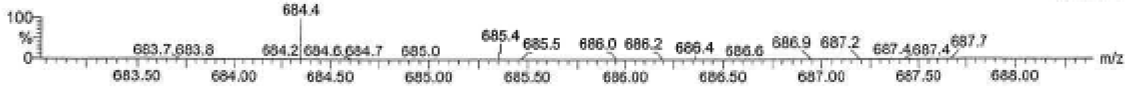


| | | | | | | |
|---|---|---|---|---|---|---|
| Minimum: | | | | -1.5 | | |
| Maximum: | | 5.0 | 10.0 | 150.0 | | |

| Mass | Calc. Mass | mDa | PPM | DBE | i-FIT | Formula |
|---|---|---|---|---|---|---|
| 684.3508 | 684.3510 | -0.2 | -0.3 | 18.5 | 2.9 | C37 H46 N7 O6 |
| | 684.3543 | -3.5 | -5.1 | 13.5 | 13.1 | C34 H50 N7 O6 S |
| | 684.3550 | -4.2 | -6.1 | 22.5 | 16.7 | C42 H46 N5 O4 |

**Figure S32:** Elemental composition report of compound **12** (HR-ESI-MS).

**Elemental Composition Report** **Page 1**

**Single Mass Analysis**
Tolerance = 10.0 PPM / DBE: min = -1.5, max = 150.0
Element prediction: Off
Number of isotope peaks used for i-FIT = 3

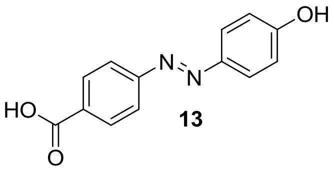

13

Monoisotopic Mass, Even Electron Ions
152 formula(e) evaluated with 1 results within limits (all results (up to 1000) for each mass)
Elements Used:
C: 0-150 H: 0-200 N: 0-6 O: 0-6

Zhang, Yagang, scz-zyg-3-22 University of Illinois, SCS, Mass Spectrometry Lab
Qtof_36536 43 (3.079) AM (Cen,3, 80.00, Ar,15000.0,734.47,0.70,LS 3); Sm (SG, 2x3.00)
Q-tof UE521
1: TOF MS ES+
6.61e+002

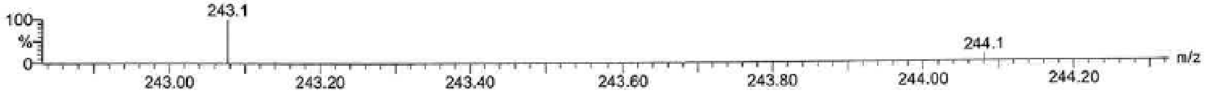


| | | | | | | |
|---|---|---|---|---|---|---|
| Minimum: | | | | -1.5 | | |
| Maximum: | | 5.0 | 10.0 | 150.0 | | |
| Mass | Calc. Mass | mDa | PPM | DBE | i-FIT | Formula |
| 243.0772 | 243.0770 | 0.2 | 0.8 | 9.5 | n/a | C13 H11 N2 O3 |

**Figure S33:** Elemental composition report of compound **13** (HR-ESI-MS).

**Elemental Composition Report** **Page 1**

**Single Mass Analysis**
Tolerance = 10.0 PPM / DBE: min = -1.5, max = 150.0
Element prediction: Off
Number of isotope peaks used for i-FIT = 3

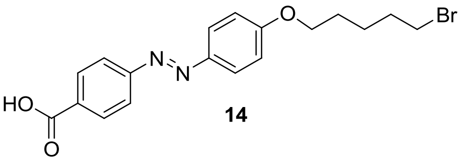

14

Monoisotopic Mass, Even Electron Ions
309 formula(e) evaluated with 3 results within limits (all results (up to 1000) for each mass)
Elements Used:
C: 0-150 H: 0-200 N: 0-5 O: 0-6 Na: 0-1 Br: 1-1

Zhang, Yagang, scz-zyg-3-24-2 University of Illinois, SCS, Mass Spectrometry Lab
Qtof_36594 48 (3.436) AM (Cen,3, 80.00, Ar,15000.0,734.47,0.70,LS 3); Sm (SG, 2x3.00); Cm (48:49)
Q-tof UE521
1: TOF MS ES+
9.75e+002

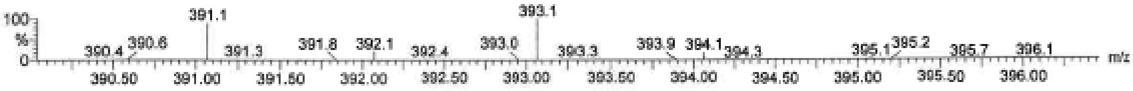


| | | | | | | |
|---|---|---|---|---|---|---|
| Minimum: | | | | -1.5 | | |
| Maximum: | | 5.0 | 10.0 | 150.0 | | |
| Mass | Calc. Mass | mDa | PPM | DBE | i-FIT | Formula |
| 391.0657 | 391.0657 | 0.0 | 0.0 | 9.5 | 2.5 | C18 H20 N2 O3 Br |
| | 391.0673 | -1.6 | -4.1 | 10.5 | 2.5 | C21 H21 O Na Br |
| | 391.0633 | 2.4 | 6.1 | 6.5 | 4.8 | C16 H21 N2 O3 Na Br |

**Figure S34:** Elemental composition report of compound **14** (HR-ESI-MS).

**Elemental Composition Report** Page 1

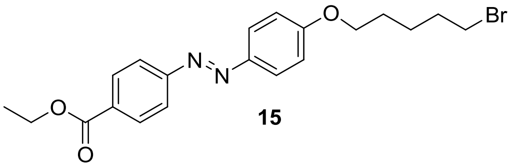


15

**Single Mass Analysis**
Tolerance = 10.0 PPM / DBE: min = -1.5, max = 150.0
Element prediction: Off
Number of isotope peaks used for i-FIT = 3

Monoisotopic Mass, Even Electron Ions
153 formula(e) evaluated with 3 results within limits (all results (up to 1000) for each mass)
Elements Used:
C: 0-150 H: 0-200 N: 0-5 O: 0-5 Br: 1-1

Zhang, Yagang, scz-zyg-3-26-1 University of Illinois, SCS, Mass Spectrometry Lab Q-tof UE521
Qtof_36632 45 (3.221) AM (Cen,3, 80.00, Ar,15000.0,734.47,0.70,LS 3); Sm (SG, 2x3.00); Cm (45) 1: TOF MS ES+
2.80e+003

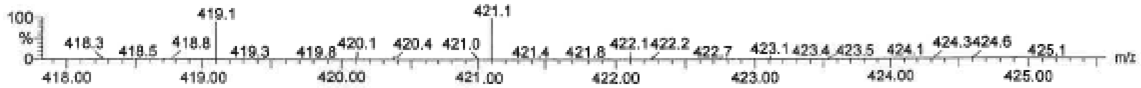


| Mass | Calc. Mass | mDa | PPM | DBE | i-FIT | Formula |
|---|---|---|---|---|---|---|
| Minimum: | | | | -1.5 | | |
| Maximum: | | 5.0 | 10.0 | 150.0 | | |
| 419.0969 | 419.0970 | -0.1 | -0.2 | 9.5 | 5.8 | C20 H24 N2 O3 Br |
| | 419.0930 | 3.9 | 9.3 | 5.5 | 15.0 | C15 H24 N4 O5 Br |
| | 419.1011 | -4.2 | -10.0 | 13.5 | 22.3 | C25 H24 O Br |

**Figure S35:** Elemental composition report of compound **15** (HR-ESI-MS).

**Elemental Composition Report** Page 1

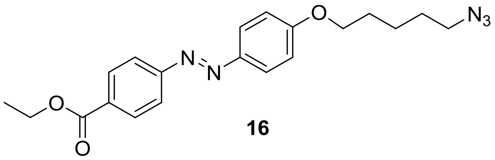


16

**Single Mass Analysis**
Tolerance = 10.0 PPM / DBE: min = -1.5, max = 150.0
Element prediction: Off
Number of isotope peaks used for i-FIT = 3

Monoisotopic Mass, Even Electron Ions
198 formula(e) evaluated with 1 results within limits (all results (up to 1000) for each mass)
Elements Used:
C: 0-150 H: 0-200 N: 0-5 O: 0-6
Zhang, Yagang, scz-zyg-3-27 University of Illinois, SCS, Mass Spectrometry Lab Q-tof UE521
Qtof_36663 32 (2.293) AM (Cen,3, 80.00, Ar,15000.0,716.46,0.70,LS 3); Sm (SG, 2x3.00); Cm (32:34) 1: TOF MS ES+
1.21e+003

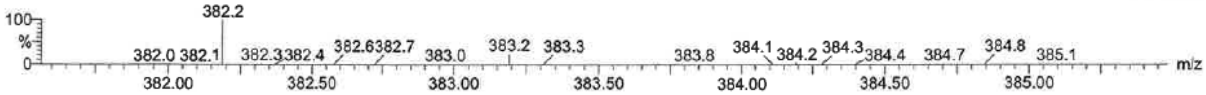


| Mass | Calc. Mass | mDa | PPM | DBE | i-FIT | Formula |
|---|---|---|---|---|---|---|
| Minimum: | | | | -1.5 | | |
| Maximum: | | 5.0 | 10.0 | 150.0 | | |
| 382.1879 | 382.1879 | 0.0 | 0.0 | 11.5 | 1.1 | C20 H24 N5 O3 |

**Figure S36:** Elemental composition report of compound **16** (HR-ESI-MS).

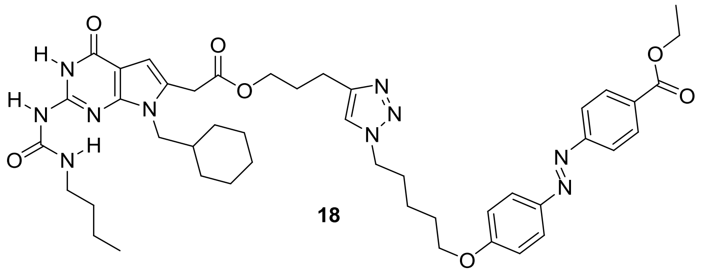


**18**

**Elemental Composition Report** **Page 1**

**Single Mass Analysis**
Tolerance = 10.0 PPM / DBE: min = -1.5, max = 150.0
Element prediction: Off
Number of isotope peaks used for i-FIT = 3

Monoisotopic Mass, Even Electron Ions
67 formula(e) evaluated with 2 results within limits (all results (up to 1000) for each mass)
Elements Used:
C: 0-150 H: 0-200 N: 10-11 O: 7-8 Na: 0-1
Zhang, Yagang, scz-zyg-3-29 University of Illinois, SCS, Mass Spectrometry Lab Q-tof UE521
Qtof_36727 37 (1.576) AM (Cen,3, 80.00, Ar,15000.0,716.46,0.70,LS 3); Sm (SG, 2x3.00); Cm (37:40) 1: TOF MS ES+
8.57e+003

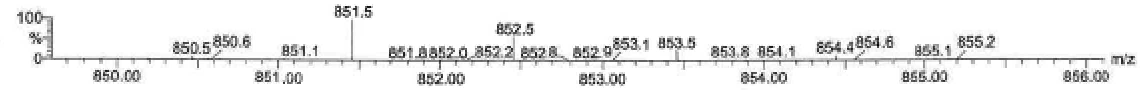


| | | | | | | |
|---|---|---|---|---|---|---|
| Minimum: | | | | -1.5 | | |
| Maximum: | | 5.0 | 10.0 | 150.0 | | |
| Mass | Calc. Mass | mDa | PPM | DBE | i-FIT | Formula |
| 851.4562 | 851.4568 | -0.6 | -0.7 | 21.5 | 125.9 | C45 H59 N10 O7 |
| | 851.4544 | 1.8 | 2.1 | 18.5 | 167.5 | C43 H60 N10 O7 Na |

**Figure S37:** Elemental composition report of compound **18** (HR-ESI-MS).

## References


1. Still, W. C.; Kahn, M.; Mitra, A. *J. Org. Chem.* **1978**, *43*, 2923–2925. doi:10.1021/jo00408a041
